# Altered Expression Patterns of Inflammation-Associated and Trophic Molecules in Substantia Nigra and Striatum Brain Samples from Parkinson's Disease, Incidental Lewy Body Disease and Normal Control Cases

**DOI:** 10.3389/fnins.2015.00507

**Published:** 2016-01-14

**Authors:** Douglas G. Walker, Lih-Fen Lue, Geidy Serrano, Charles H. Adler, John N. Caviness, Lucia I. Sue, Thomas G. Beach

**Affiliations:** ^1^Banner Sun Health Research InstituteSun City, AZ, USA; ^2^Neurodegenerative Disease Research Center, Biodesign Institute, Arizona State UniversityTempe, AZ, USA; ^3^Neurology, Mayo Clinic College of MedicineScottsdale, AZ, USA

**Keywords:** inflammation, pathology, cytokines, antibody array, dopaminergic cell loss, Parkinson's disease, microglia, astrocytes

## Abstract

Evidence of inflammation has been consistently associated with pathology in Parkinson's disease (PD)-affected brains, and has been suggested as a causative factor. Dopaminergic neurons in the substantia nigra (SN) pars compacta, whose loss results in the clinical symptoms associated with PD, are particularly susceptible to inflammatory damage and oxidative stress. Inflammation in the striatum, where SN dopaminergic neurons project, is also a feature of PD brains. It is not known whether inflammatory changes occur first in striatum or SN. Many animal models of PD have implicated certain inflammatory molecules with dopaminergic cell neuronal loss; however, there have been few studies to validate these findings by measuring the levels of these and other inflammatory factors in human PD brain samples. This study also included samples from incidental Lewy body disease (ILBD) cases, since ILBD is considered a non-symptomatic precursor to PD, with subjects having significant loss of tyrosine hydroxylase-producing neurons. We hypothesized that there may be a progressive change in key inflammatory factors in ILBD samples intermediate between neurologically normal and PD. To address this, we used a quantitative antibody-array platform (Raybiotech-Quantibody arrays) to measure the levels of 160 different inflammation-associated cytokines, chemokines, growth factors, and related molecules in extracts of SN and striatum from clinically and neuropathologically characterized PD, ILBD, and normal control cases. Patterns of changes in inflammation and related molecules were distinctly different between SN and striatum. Our results showed significantly different levels of interleukin (IL)-5, IL-15, monokine induced by gamma interferon, and IL-6 soluble receptor in SN between disease groups. A different panel of 13 proteins with significant changes in striatum, with IL-15 as the common feature, was identified. Although the ability to detect some proteins was limited by sensitivity, patterns of expression indicated involvement of certain T-cell cytokines, vascular changes, and loss of certain growth factors, with disease progression. The results demonstrate the feasibility of profiling inflammatory molecules using diseased human brain samples, and have provided additional targets to validate in relation to PD pathology.

## Introduction

Parkinson's disease (PD) is a chronic, progressive neurodegenerative disorder characterized by symptoms of tremor, bradykinesia, ataxia and rigidity, and is the main cause of movement disorders in the elderly. The current estimates of PD are one million cases in the U.S.A. with 60,000 cases being added each year (Parkinson's Disease Foundaton, 2015). This leads to significant morbidity and mortality in affected individuals along with the considerable cost of healthcare for PD patients. Sufferers of PD also have significant risk of progressing to dementia (Aarsland et al., 2005). There have been major strides in understanding the cause(s) of PD, but there is still lack of consensus of the sequence of events that lead to the loss of the dopaminergic neurons of the substantia nigra (SN) pars compacta and results in loss of dopaminergic neurotransmission in the striatum. Much research on disease mechanisms has focused on the properties of the presynaptic vesicle protein α-synuclein whose modifications can lead to the formation of aggregated and neurotoxic species (Vekrellis and Stefanis, 2012; Lawand et al., 2015; Osterberg et al., 2015; Sian-Hulsmann et al., 2015). Aggregated α-synuclein, particularly the phosphorylated form, is the major component of Lewy bodies, the major pathological feature of PD brains (Fujiwara et al., 2002). Most of the identified mutations in α-synuclein appear to increase the risk of PD by increasing its tendency to aggregate (Polymeropoulos, 1997; Baba et al., 1998; Conway et al., 1998; Ancolio et al., 2000; Ostrerova-Golts et al., 2000; Ghosh et al., 2013; Giráldez-Pérez et al., 2014).

Inflammation has also been a well-established feature of PD pathology (McGeer et al., 1988; Boka et al., 1994; Mogi et al., 1994; Hunot et al., 1999; Brochard et al., 2009), and some data have suggested it could be the primary pathological cause for SN neuronal cell death (Tansey et al., 2007, 2008; Vivekanantham et al., 2015). Inflammation in PD brains could be caused by different factors, but a number of studies have shown that aggregated forms of α-synuclein can activate microglia to produce toxic molecules that contribute to dopaminergic cell death (Zhang et al., 2005; Couch et al., 2011; Béraud et al., 2013; Acosta et al., 2015). Alpha-synuclein activation of microglia can be mediated through the Toll-like receptor (TLR)-2 (Codolo et al., 2013; Kim et al., 2013; Doorn et al., 2014; Daniele et al., 2015), though other microglial receptors have also been implicated, including TLR-4 (Stefanova et al., 2011; Fellner et al., 2013) and purinergic receptor P2X7 (Jiang et al., 2015). A recent immunohistochemical study of SN from control, ILBD and PD cases for the inflammation marker TLR-2 showed increased numbers of TLR-2-positive microglia in ILBD cases compared to PD cases (Doorn et al., 2014). This suggested that some inflammatory changes could be happening at early stages prior to development of symptoms of PD. By contrast, there was progressive increase from control to PD in numbers of CD68-positive amoeboid microglia/macrophages, a marker associated with phagocytosis, which correlated with an increase in α-synuclein deposits (Doorn et al., 2014). Independent of the presence of pathological α-synuclein, the human SN appears to be particularly sensitive to inflammation, possibly due to higher concentrations of iron, and neuromelanin, both of which can contribute to an environment of enhanced oxidative stress (Hirsch, 1993; Béraud et al., 2013; Taylor et al., 2013; Fischer and Maier, 2015). Purified neuromelanin can directly activate microglia to a proinflammatory state (Wilms et al., 2003; Zhang et al., 2013).

Animal models for PD have provided the most convincing evidence for how inflammation could be directly linked to SN neuronal cell loss. A widely used model involves the administration of the bacterial cell wall extract lipopolysaccharide (LPS) (Couch et al., 2011; Qin et al., 2013; Tanaka et al., 2013; Sharma and Nehru, 2015). Both direct injection of LPS into the SN or by intraperitoneal injection can lead to enhanced inflammation in the brain and selective loss of SN neurons. Administration of LPS results in increased production of free radicals and potentially toxic cytokines, including tumor necrosis factor-α (TNF-α) (McGuire et al., 2001; Pei et al., 2007; Tansey et al., 2007, 2008; Zhao et al., 2007; Gao et al., 2011; Tran et al., 2011; Montgomery and Bowers, 2012; Qin et al., 2013). Another feature of inflammation-induced models of PD is disruption of the blood-brain barrier, which enhances inflammation by permitting influx of components of cell-mediated immunity (Carvey et al., 2005b, 2006; Desai et al., 2007; Monahan et al., 2008).

The spread of abnormal forms of α-synuclein along neuroanatomical pathways is a significant pathological mechanism in humans and animal models of synucleinopathies (Beach et al., 2009; Luk et al., 2012; Masuda-Suzukake et al., 2014; Paumier et al., 2015). In animal models, instrastriatal injection of preformed α-synuclein fibrils resulted in neurodegeneration and inflammation in the SN; the reverse effect occurs with α-synuclein administered into the SN resulting in striatum pathology and inflammation (Koprich et al., 2010; Luk et al., 2012; Osterberg et al., 2015; Paumier et al., 2015). Direct injection of LPS into striatum or SN resulted in degeneration of nigrostriatal pathway neurons and motor impairments, along with microglial activation (Choi et al., 2009; Couch et al., 2011). Co-administration of α-synuclein and LPS can significantly enhance the generation of nigrostriatal pathology (Couch et al., 2011; Gao et al., 2011). As it is still not known if spread of pathology or neuroinflammation occurs from striatum to SN or vice-versa, as part of this study, we sought to compare the changes in inflammation and trophic molecules between SN and striatum with disease progression. Recent mouse models of PD have focused on α-synuclein, either overexpression of normal or mutated α-synuclein under transgene control or overexpression by administration of α-synuclein viral transduction vectors (Watson et al., 2012; Béraud et al., 2013; Gardai et al., 2013; Harms et al., 2013). Animal models of PD can also be developed using the dopaminergic neurotoxin 1-methyl-4 phenyl-1,2,3,6-tetrahydropyridine (MPTP) to lesion the SN and striatum. This model can reproduce many PD features in non-human primates (Ohnuki et al., 2010). This study carried out gene expression profiling of SN and striatum tissue and showed significant downregulation of neuronal and dopaminergic genes in lesioned animals. With respect to inflammation-related genes, only upregulation of glial fibrillary acidic protein (GFAP), interleukin (IL)-11, and chemokines CXCL13 and CXCL4 were detected (Ohnuki et al., 2010).

Although there have been many immunohistological studies that used antibodies to activated microglia to demonstrate inflammation in PD brains, there have been few studies that biochemically measured levels of cytokines or other inflammation-associated molecules in human brain samples (Nagatsu et al., 2000a,b). A recent proteomics analysis comparing SN tissue from PD and control cases identified cytosolic non-specific dipeptidase 2 to be upregulated in PD tissue, but not inflammatory or growth factor molecules. This may have been due to lack of sensitivity of the methods employed to detect these low-abundance molecules (Licker et al., 2012). Such experiments are technically difficult as only low concentrations of key molecules are present in tissue. Cerebrospinal fluid (CSF), plasma and blood have also been used as surrogates to follow brain changes in PD (Rocha et al., 2014), but these results have generally been inconsistent between studies. Increased levels of IL-2, IL-6, and TNF-α have been detected in CSF samples of PD subjects compared to controls (Mogi et al., 1994, 1996). Screening of sera from control, PD, multiple system atrophy and corticobasal syndrome cases using a similar antibody array as used in this study, showed only platelet-derived growth factor (PDGF)-BB and prolactin having significant disease associated differences (Mahlknecht et al., 2012). Measurement of cytokines in CSF from PD and control cases identified significant changes in the cytokines/growth factors vascular endothelial growth factor (VEGF), placental growth factor (PIGF), soluble VEGF receptor (sVEGFR2), and angiopoietin2 (ANG2), associated with angiogenesis (Janelidze et al., 2015).

To address the problems of sensitivity, studies have used mRNA gene expression profiling of PD SN tissue, or laser-dissected SN dopaminergic neurons, to identify disease differences. These techniques have high sensitivity for detecting low-abundance gene expression, but there has been lack of consensus on the PD-associated differentially expressed genes between studies. However, these studies have identified multiple pathways affected in PD, with downregulation of genes associated with synaptic function, cytoskeletal function and neuroprotection and also ubiquitin-proteosome and mitochondrial function genes being features (Grünblatt et al., 2004; Hauser et al., 2005; Mandel et al., 2005; Duke et al., 2006; Elstner et al., 2009; Ohnuki et al., 2010; Gründemann et al., 2011; Botta-Orfila et al., 2012). A microarray study focusing on inflammatory gene expression in PD SN showed upregulation of the microglial purinergic receptor P2X7 (a receptor for ATP), colony stimulating factor-1 receptor (CSF1R), (a microglia growth factor receptor), and nitric oxide synthase 3 (a vascular marker) (Durrenberger et al., 2012).

With the development of high-sensitivity multiplex antibody arrays and other proteomic techniques, it is possible to profile large numbers of different biologically-active proteins in human brain tissue or other biological samples. We used this approach to examine the levels of 158 proteins in SN and striatum of control, ILBD and PD cases to determine if there were progressive changes in inflammation or related proteins. We particularly sought to determine if any of the cytokines identified in PD animal models could be validated in these human tissues. Our results demonstrated distinctly different patterns of inflammation and growth factor changes between SN and striatum with disease.

## Materials and methods

### Brain tissue samples

Brain tissue samples for this study were provided by the Banner Sun Health Research Institute Brain and Body Donation Program. The Brain and Body Donation Program operated with the approval of Western IRB (Puyallup, WA) under contract as the Institutional Review Board of Banner Research. A summary of the demographics of the cases is shown in Table 1. There were SN samples from 16 controls, 21 ILBD, and 18 PD cases; and striatum samples from 16 controls, 17 ILBD, and 16 PD cases. Tissue samples from both SN and striatum were not available for all cases—overlap of cases between brain regions was 92%. The selection of control, ILBD and PD cases used in this study was based on neuropathology diagnosis with reference to clinical records, with a diagnosis of Alzheimer's disease (AD) as the principal exclusion criteria. The degree of Lewy body (LB) pathology was assessed using a histological staging scheme in each of 10 brain regions (Beach et al., 2009). This involved obtaining a ranking score (0-4) using phosphorylated-alpha synuclein stained sections from each region. These numbers are summed to give Lewy body pathology scores of 0-40 for each brain. Histological ranking scores of plaques and tangles in five brain regions were used to assess how much age-associated AD-type pathology was present in each case (Beach et al., 2012; Table 1). This involved obtaining a ranking score (0–3) using Thioflavin S-stained tissue sections from entorhinal cortex, hippocampus, frontal, parietal, and temporal cortex. These numbers are summed to give plaque and tangle scores of 0–15 for each brain. Dementia was present in a number of the PD cases (Table 1); however, neuropathology and clinical records indicated this was not due to AD.

**Table 1:** **Demographic features of cases used in study**.

**Disease Type**	***n***	**Age (Mean + SD)**	**Sex M/F**	**Dementia (D/MCI/CN)**	**LB**	**Plaques (Mean + SD)**	**Tangles (Mean + SD)**
**SUBSTANTIA NIGRA**							
Control	16	81.2 ± 11.4	9/7	0/16	0	1.4 ± 1.8	2.4 ± 1.6
ILBD	21	86.8 ± 6.8	17/4	0/3/18	1-3	3.5 ± 4.6	3.9 ± 2.2
PD (DD 12.9; 1–26)	18	81.3 ± 6.2	12/6	7/3/8	2b-4	3.2+3.6	3.9 ± 2.1
**STRIATUM**							
Control	16	81.4 ± 11.5	10/6	0/16	0	1.5 ± 1.9	2.5 ± 1.8
ILBD	17	85.2 ± 6.4	13/4	0/2/15	1–3	3.4 ± 3.8	3.2 ± 1.3
PD (DD 13.4; 1–26)	16	81.3 ± 6.2	12/6	7/2/7	2b–4	3.2+3.6	4.1 ± 2.1

*ILBD, incidental Lewy body disease; PD, Parkinson's disease; n, number of cases; M/F, male/female; LB, Lewy body staging score; Plaques, Plaque score; Tangles, tangle score; D, demented; MCI, mild cognitive impairment; CN, cognitively normal; DD, disease duration/years—mean and range*.

### Tissue preparation

Due to the limited amount of SN tissue available from each case, SN tissue was provided as 15 consecutive frozen (10 μm) SN sections (~6–10 mg) cut from each block with a cryostat and collected frozen. This approach to tissue sample preparation allowed the selection of matched samples containing approximately equivalent densities of SN pars compacta neuromelanin-containing neurons. Frozen striatum samples (20–30 mg) were dissected from putamen at the level of the lenticular nucleus.

### Processing of tissue samples

Both series of tissue samples were extracted in six volumes (w/v) of a proprietary extraction buffer compatible with the Quantibody arrays (Raybiotech, Norcross, GA) supplemented with protease/phosphatase inhibitors (Thermo-Fisher/Pierce). Samples were briefly sonicated in extraction buffer and incubated on ice with constant shaking for 30 min. After centrifugation (18,000 g/30 min), the supernatants were collected for further analysis. Protein concentrations were determined using a micro BCA assay (Thermo-Fisher/Pierce). These same extracts were also used to prepare western blot samples.

### Tissue analyses

Preliminary analyses of protein extracts were carried out using western blot methods for tyrosine hydroxylase (TH), as a measure of degree of dopaminergic neuron cell loss; for IBA-1, an indicator of microglia abundance; for TLR-2 as a marker for inflammation; and for glial fibrillary acidic protein (GFAP), as an indicator of reactive astrocytosis. We employed our standard published western blot methods using antibodies to TH (Biolegend, Dedham, MA; rabbit polyclonal 1:2000 dilution); to IBA-1 (Wako Chemicals, Richmond, VA, 1:2000); to TLR-2 (Abcam, Cambridge, MA; rabbit monoclonal 1:2500 dilution); and to GFAP [BD Biosciences, Franklin Lakes, NJ; cocktail of three different monoclonal antibodies (1:2000)], Walker et al., 2015a,b.

For the antibody array analysis, samples were processed as a service by Raybiotech (Norcross, GA). For SN, each sample protein concentration was adjusted to 500 μg/ml, and for striatum, each sample was adjusted to 1000 μg/ml. These were analyzed using 160 protein Quantibody arrays (catalog number QAH-CAA-3000) composed of antibody-coated glass slide arrays for detecting cytokines, chemokines, soluble receptor, and growth factors. Table 2 shows the arrangement of the proteins on the four separate array slides. Each protein had a standard curve included with measurements from known dilutions of purified standard protein. Each slide array contained positive control samples that were used for normalization purposes. Measurements were based on fluorescent intensity of bound labeled antibodies to each spot, and calculated from the mean of four spots/antibody. Slides were measured, analyzed, and normalized to positive controls using Raybiotech software. Final results were expressed as pg of protein/ml extract.

**Table 2:** **Quantibody array component proteins**.

**Array-CHE**	**Array-CYT**	**Array-REC**	**Array-GF**
6Ckine	BLC	4-1BB	AR
Axl	Eotaxin	ALCAM	BDNF
BTC	Eotaxin-2	B7-1	bFGF
CCL28	G-CSF	BCMA	BMP-4
CTACK	GM-CSF	CD14	BMP-5
CXCL16	I-309	CD30	BMP-7
ENA-78	ICAM-1	CD40 L	b-NGF
Eotaxin-3	IFNγ	CEACAM-1	EGF
GCP-2	IL-1α	DR6	EGF-R
GRO	IL-1β	Dtk	EG-VEGF
HCC-1	IL-1ra	Endoglin	FGF-4
HCC-4	IL-2	ErbB3	FGF-7
IL-9	IL-4	E-Selectin	GDF-15
IL-17F	IL-5	Fas	GDNF
IL-18 BPa	IL-6	Flt-3L	GH
IL-28A	IL-6sR	GITR	HB-EGF
IL-29	IL-7	HVEM	HGF
IL-31	IL-8	ICAM-3	IGFBP-1
IP-10	IL-10	IL-1 R4	IGFBP-2
I-TAC	IL-11	IL-1 RI	IGFBP-3
LIF	IL-12p40	IL-2 Rg	IGFBP-4
LIGHT	IL-12p70	IL-10 Rb	IGFBP-6
Ltactin	IL-13	IL-17R	IGF-I
MCP-2	IL-15	IL-21R	Insulin
MCP-3	IL-16	LIMPII	MCF R
MCP-4	IL-17	Lipocalin-2	NGF R
MDC	MCP-1	L-Selectin	NT-3
MIF	MCSF	LYVE-1	NT-4
MIP-3a	MIG	MICA	OPG
MIP-3b	MIP-1a	MICB	PDGF-AA
MPIF-1	MIP-1b	NRG1-b1	PIGF
MSPa	MIP-1d	PDGF Rb	SCF
NAP-2	PDGF-BB	PECAM-1	SCF R
OPN	RANTES	RAGE	TGFa
PARC	TIMP-1	TIM-1	TGFb1
PF4	TIMP-2	TRAIL R3	TGFb3
SDF-1a	TNFa	Trappin-2	VEGF
TARC	TNFb	uPAR	VEGF R2
TECK	TNF RI	VCAM-1	VEGF R3
TSLP	TNF RII	XEDAR	VEGF-D

*Abbreviations/Definitions: Supplemental Table 1*.

### Immunohistochemical staining of SN and striatum tissue sections

To characterize features of microglia activation in tissues being examined by antibody array analysis, 40μm paraformaldehyde-fixed tissue sections of SN and striatum from control, ILBD and PD cases were stained using antibodies LN3 (Abcam, Cambridge, MA) and IBA-1 (Wako Chemicals, Richmond, VA). These are well-established markers for activated microglia (LN3) or pan microglia (IBA-1). Striatum sections were double-stained for TH (Biolegend—rabbit polyclonal, 1:2000) using a two-color method. Immunohistochemstry was carried out according to our published procedures (Walker et al., 2009, 2015b).

### Data analysis

The validity of the standard curve for each protein was confirmed by visual inspection of data. We found that standard curves for basic fibroblast growth factor and insulin growth factor binding protein-4 were unsatisfactory as the standard proteins did not produce a suitable dose-response curve; the results obtained for these proteins in tissue samples were excluded.

Data for each brain region samples were grouped as control, ILBD and PD and analyzed by One-way analysis of variance (ANOVA) using Graphpad Prism 5 software (Graphpad software, La Jolla, CA) without corrections for multiple comparisons. To determine whether age of patient or postmortem interval (PMI) were covariants for each measure, data from each protein was also analyzed by Analysis of Covariance (ANCOVA) with age, PMI, or age and PMI as covariants using Medcalc statistical software (Medcalc Software, Ostend, Belgium). Further analyses included correlation analyses, stepwise logistic regression analyses and receiver operating characteristic (ROC) curve analyses and were carried out using MedCalc. For each measure, *P* < 0.05 was considered statistically significant.

## Results

### Characterization of samples

**Figure 1:**
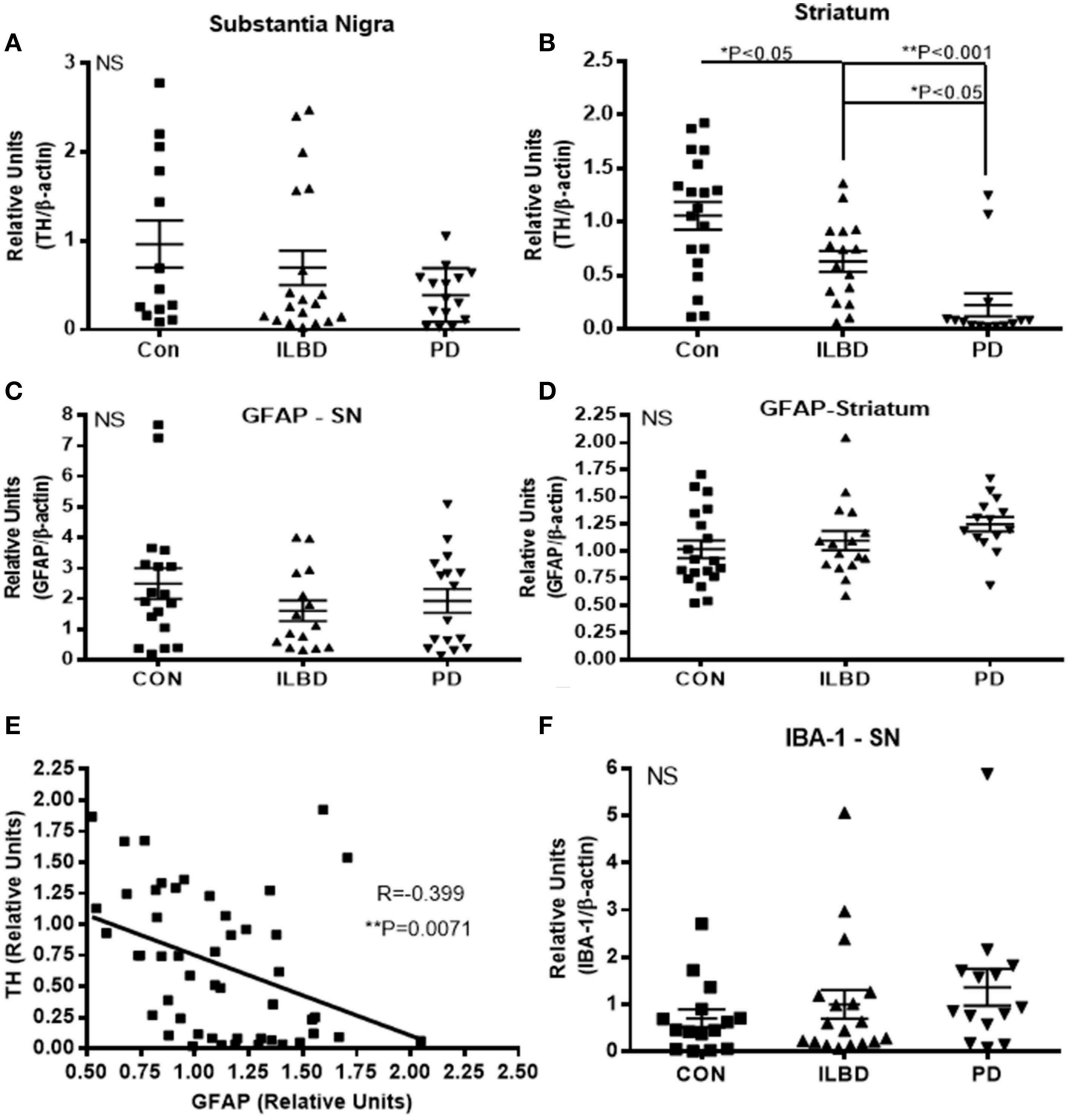
**Relative levels of tyrosine hydroxylase, glial fibrillary acidic protein or IBA-1 in substantia nigra**. **(A,B)** Relative levels of tyrosine hydroxylase (TH) in control (Con), incidental Lewy body disease (ILBD), and Parkinson's disease (PD) samples of SN **(A)** or striatum **(B)** determined by western blot measures of TH with normalization for levels of β-actin. Statistical analysis by One-way analysis of variance (ANOVA) with Fisher LSD *post-hoc* test for between group differences. **(C,D)** Relative levels of glial fibrillary acidic protein (GFAP) in control (Con), incidental Lewy body disease (ILBD), and Parkinson's disease (PD) samples of SN **(C)** or striatum **(D)** determined by western blot measures of GFAP with normalization for levels of β-actin. Statistical analysis by One-way analysis of variance (ANOVA) showed no significance between disease groups. **(E)** Linear regression plot showing relation between striatum TH and striatum GFAP levels. Pearson correlation analysis showed significance between these measures (*R* = −0.399, *P* = 0.0071). **(F)** Relative levels of IBA-1 in control (Con), incidental Lewy body disease (ILBD), and Parkinson's disease (PD) samples of SN determined by western blot measures of with normalization for levels of β-actin. Statistical analysis by One-way analysis of variance (ANOVA) showed no significant difference between disease groups.

The samples were selected based on consensus clinical and neuropathological criteria, but as shown in Table 1, there were different amounts of age-associated pathology in the samples. The control samples were free of LB pathology; while the ILBD and PD cases had varying degrees (Table 1). To support the clinical and neuropathological criteria used for case selection, samples were characterized for TH levels as an additional index of disease severity. There was significant variability between the samples in each disease group for TH, especially within the control groups (Figure 1). In SN, disease group differences in TH levels did not reach statistical significance by One-way ANOVA (Figure 1A), while in striatum, expected TH differences between each of the disease groups were shown (Figure 1B). Age and PMI were not significant covariant factors affecting TH levels in SN or striatum. Pathological variability within the disease groups was also highlighted by measures of gliosis and inflammation. GFAP levels showed no significant differences between disease groups for SN (Figure 1C) or striatum (Figure 1D) samples; however, GFAP levels in striatum were significantly affected by PMI (*P* = 0.03). There was significant negative correlation between TH and GFAP levels in striatum (Pearson *r* = −0.399, *P* = 0.0071; Figure 1E) suggesting increased gliosis as PD pathology progresses. Western blots measures of the microglial marker IBA-1 in SN (Figure 1F) and striatum (data not shown) did not show significant disease group differences; these measures were not affected by age or PMI.

### Inflammatory profiling of SN and striatum

Initial data analysis of results from the Quantibody arrays consisted of individual ANOVA for each protein measure between the three disease groups for each of the brain regions. Results are shown in Table 3, which lists all *P*-values between groups for each protein. These results were not corrected for multiple comparisons. Table 3 also identifies the proteins that gave negative values for each brain region. A negative designation was applied when fewer than 10 samples out of the total analyzed were above the limit of detection (LOD). For striatum samples, 57 of the 158 proteins measured were considered negative, while for SN, the same 57 were negative along with an additional 20 proteins (Table 3).

**Table 3:** **Summary of One-way ANOVA results**.

**Array**			**Array**			**Array**			**Array**		
**CHE**	**SN**	**Striatum**	**CYTO**	**SN**	**Striatum**	**REC**	**SN**	**Striatumm**	**GF**	**SN**	**Striatum**
6Ckine	Neg	Neg	BLC	Neg	Neg	4-1BB	Neg	Neg	AR	0.76	0.756
Axl	0.179	0.643	Eota xin	Neg	Neg	ALCAM	0.836	0.527	BDNF	0.295	0.709
BTC	Neg	Neg	Eotax in-2	Neg	Neg	B7-1	Neg	Neg	*bFGF*	*Reject*	*Reject*
CCL28	0.503	**0.013**	G-CSF	Neg	Neg	BCMA	Neg	Neg	BMP-4	0.349	0.671
CTACK	Neg	Neg	GM-CSF	Neg	Neg	CD14	0.928	0.521	BMP-5	Neg	Neg
CXCL16	0.478	0.352	I-309	Neg	Neg	CD30	Neg	Neg	BMP-7	Neg	Neg
ENA-78	Neg	0.156	ICAM-1	0.621	0.83	CD40 L	Neg	Neg	b-NGF	Neg	0.867
Eotaxin-3	Neg	Neg	IFNg	0.832	**0.044**	CEACAM-1	0.941	0.655	EGF	0.406	0.79
GCP-2	Neg	Neg	IL-1α	Neg	Neg	DR6	Neg	0.967	EGF-R	0.77	0.483
GRO	0.575	0.289	IL-1β	Neg	Neg	Dtk	0.539	**0.031**	EG-VEGF	0.465	0.049
HCC-1	0.336	**0.045**	IL-1ra	0.671	**0.002**	Endoglin	0.935	0.299	FGF-4	Neg	Neg
HCC-4	Neg	Neg	IL-2	0.244	**0.028**	ErbB3	0.252	**0.004**	FGF-7	Neg	0.948
IL-9	Neg	Neg	IL-4	Neg	0.103	E-Selectin	0.496	0.582	GDF-15	0.218	0.401
IL-17F	Neg	Neg	IL-5	**0.035**	0.132	Fas	0.631	0.772	GDNF	0.766	0.394
IL-18 BPa	0.822	**0.015**	IL-6	0.219	0.067	Flt-3L	0.965	0.63	GH	0.663	0.397
IL-28A	Neg	Neg	IL-6sR	**0.022**	0.187	GITR	0.435	0.669	HB-EGF	Neg	0.83
IL-29	Neg	Neg	IL-7	0.631	0.146	HVEM	0.357	0.669	HGF	0.426	0.274
IL-31	Neg	Neg	IL-8	0.272	0.154	ICAM-3	0.103	0.073	IGFBP-1	0.553	0.192
IP-10	Neg	0.41	IL-10	Neg	Neg	IL-1 R4	Neg	0.277	IGFBP-2	0.731	0.316
I-TAC	Neg	0.91	IL-11	Neg	Neg	IL-1 RI	Neg	0.839	IGFBP-3	0.147	0.639
LIF	Neg	Neg	IL-12p40	Neg	Neg	IL-2 Rg	Neg	0.72	IGFBP-4	*Reject*	*Reject*
LIGHT	Neg	Neg	IL-12p70	Neg	Neg	IL-10 Rb	0.991	0.344	IGFBP-6	0.565	0.783
Ltactin	0.788	0.723	IL-13	0.231	0.091	IL-17R	0.345	0.336	IGF-I	0.097	0.391
MCP-2	0.524	0.482	IL-15	**0.033**	**0.009**	IL-21R	0.319	0.669	Insulin	Neg	Neg
MCP-3	0.887	0.753	IL-16	0.719	0.092	LIMPII	0.996	0.541	MCF R	0.389	0.32
MCP-4	Neg	Neg	IL-17	Neg	Neg	Lipoca lin-2	0.795	0.253	NGF R	0.183	0.309
MDC	Neg	Neg	MCP-1	0.081	0.295	L-Selectin	0.669	0.827	NT-3	Neg	Neg
MIF	0.883	0.477	MCSF	0.105	0.513	LYVE-1	0.781	0.988	NT-4	Neg	0.442
MIP-3a	Neg	0.544	MIG	**0.017**	0.389	MICA	0.809	0.422	OPG	Neg	0.363
MIP-3b	Neg	Neg	MIP-1a	Neg	Neg	MICB	Neg	Neg	PDGF-AA	0.14	**0.002**
MPIF-1	Neg	Neg	MIP-1b	Neg	Neg	NRG1-b1	Neg	Neg	PIGF	Neg	Neg
MSPa	Neg	Neg	MIP-1d	Neg	Neg	PDGF Rb	0.154	0.999	SCF	Neg	0.641
NAP-2	0.52	0.178	PDGF-BB	0.078	**0.003**	PECAM-1	0.394	0.184	SCF R	0.748	0.89
OPN	0.579	0.558	RANTES	0.65	0.109	RAGE	Neg	0.911	TGFa	Neg	Neg
PARC	Neg	Neg	TIMP-1	0.599	0.243	TIM-1	Neg	0.871	TGFb1	Neg	Neg
PF4	0.829	**0.02**	TIMP-2	0.184	0.08	TRAIL R3	0.754	0.839	TGFb3	Neg	Neg
SDF-1a	Neg	Neg	TNFa	0.144	**0.008**	Tra ppin-2	Neg	0.119	VEGF	Neg	0.999
TARC	Neg	Neg	TNFb	Neg	Neg	uPAR	0.078	0.533	VEGF R2	Neg	0.775
TECK	Neg	Neg	TNF RI	0.179	0.545	VCAM-1	0.432	0.21	VEGF R3	Neg	0.21
TSLP	Neg	Neg	TNF RII	0.508	0.517	XEDAR	Neg	Neg	VEGF-D	Neg	0.814

*Results represent P-values from One-way analysis of variance for the comparison of values between Control, ILBD, and PD disease groups. Neg means the protein was deemed not to be present in sufficient number of the samples (we assessed this as < 10 positive samples out of the 55 SN or 52 striatum). Measures with P values < 0.05 are highlighted in bold*.

### Protein profile of SN

**Figure 2:**
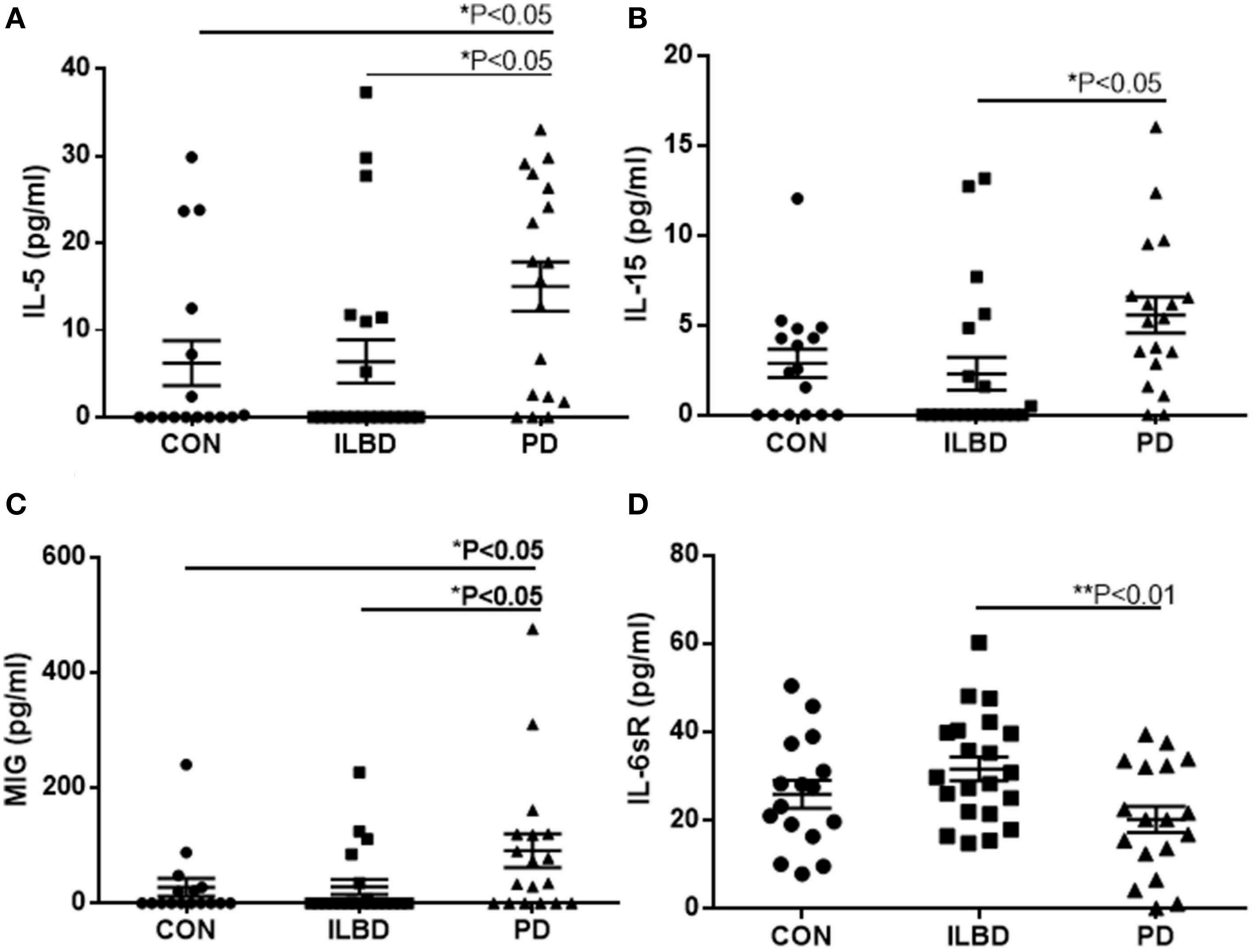
**Scatter plots showing distribution of results for proteins that showed disease group differences in substantia nigra samples**. Disease group differences for interleukin-5 (IL-5) **(A)**, IL-15 **(B)**, monokine induced by gamma interferon (MIG) **(C)**, and IL-6 soluble receptor (IL-6sR) **(D)**. Statistical analysis by One-way analysis of variance (ANOVA) with Fisher LSD *post-hoc* test for between group differences.

There were four proteins [IL-5, IL-15, monokine induced by gamma interferon (MIG) and IL-6 soluble receptor (IL6sR)] that gave *P* < 0.05 values between disease groups. The individual results for these proteins are shown as scatter plots in Figure 2. There were increases of IL-5, IL-15, and MIG, and a decrease of IL-6sR, in PD samples compared to controls or ILBD samples. Disease group differences did not become significantly different for any of these measures when corrected for age or PMI by ANCOVA; however, this analysis showed that IL-15 levels were significantly affected by PMI (ANCOVA—*P* = 0.040). In addition, when platelet derived growth factor (PDGF)-BB levels were corrected for age (*P* = 0.031—ANCOVA), a significant disease group difference was obtained (*P* = 0.0323 between ILBD and PD samples).

Stepwise logistic regression analysis of these four cytokines was carried out to determine if these measures had predictive value between disease groups. Comparing control to PD indicated that only IL-5 values had significant predictive value (*P* = 0.003) with ROC curve analysis for IL-5 with sensitivity of 85.7% and specificity of 62.5% with area under curve (AUC) of 0.81. Further stepwise logistic regression analysis between ILBD and PD values showed a combination of IL-15 and IL-6sR levels gave predictive value (*P* = 0.0016), with ROC AUC of 0.820, and sensitivity of 85.7% and specificity of 64.7%. These stepwise regression analysis models were not strengthened by inclusion of TH values. Results are summarized in Table 4. Multiple stepwise regression analyses for all measured proteins in SN showed no additional proteins or group of proteins could discriminate between control and ILBD cases, or between ILBD and PD cases (data not shown).

**Table 4:** **Sensitivity and specificity for discriminating between disease groups**.

**Measures**	**Disease groups**	**% Sensitivity (95% CI)**	**% Specificity (95% CI)**	**Area under curve**	***P***
(SN) IL-5	Control/PD	75	61.1	0.73	0.026
(SN) IL-15, IL-6sR	ILBD/PD	85.7	62.5	0.82	0.0016
(Str) ErbB3, IL-2, PF4	Control/PD	86.7	87.5	0.92	0.0001
(Str) TH	Control/PD	80	85.7	0.91	0.0001
(Str) TH, CCL28	Control/PD	80	78.7	0.94	<0.0001
(Str) PDGF-AA	ILBD/PD	72.2	62.5	0.72	0.015
(Str) TH, IL-15	ILBD/PD	82.3	85.7	0.90	0.0003
(Str) PDGF-AA	Control/ILBD	66.7	77.8	0.81	0.0014
(Str) TH	Control/ILBD	60	70.6	0.737	0.0157

### Protein profile of striatum

**Figure 3:**
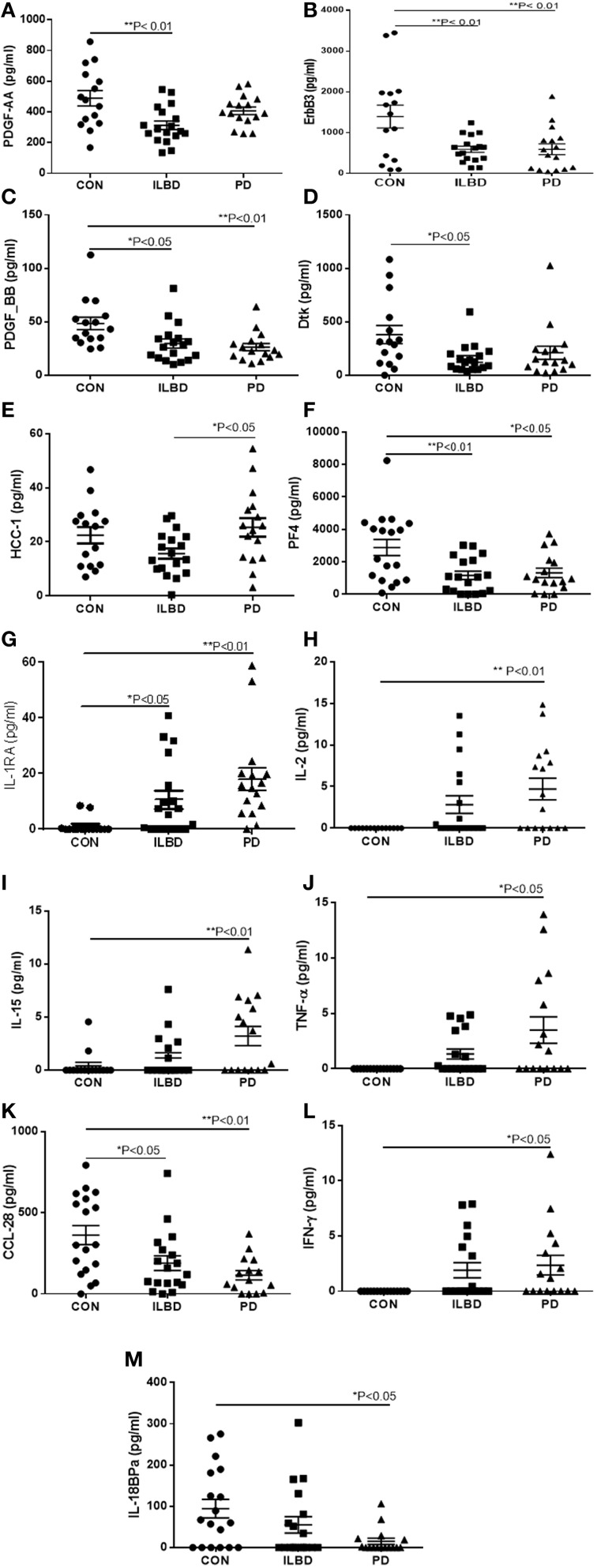
**Scatter plots showing distribution of results for proteins that showed disease group differences in striatum samples**. Disease group differences for platelet derived growth factor-AA (PDGF-AA) **(A)**, ErbB3 **(B)**, PDGF-BB **(C)**, and Dtk **(D)**. HCC-1, **(E)**, platelet factor-4 (PF4) **(F)**, IL-1 receptor antagonist (IL-1RA) **(G)**, IL-2 **(H)**, IL-15 **(I)**, TNF-α **(J)**, CCL-28 **(K)**, IFN-γ **(L)**, IL-18 binding protein (IL-18bpa) **(M)**. Statistical analysis by One-way analysis of variance (ANOVA) with Fisher LSD *post-hoc* test for between group differences.

A panel of 13 proteins was shown to have significant differences in striatum samples between the disease groups by One-way ANOVA (Table 3)—CCL28 (C–C motif ligand 28 or mucosae-associated epithelial chemokine), HCC-1 (CCL-14), IL-18 binding protein a (IL-18BPa), PF4 (platelet factor 4), interferon-gamma (IFN-γ), IL-1 receptor antagonist (IL-1ra), IL-2, IL-15, PDGF-AA, PDGF-BB, TNF-α, Dtk (tyrosine-protein kinase receptor TYRO3), and ErbB3 (also HER3, human epidermal growth factor receptor 3). The individual scatter plot results for these proteins are shown in Figure 3. When correcting values for age, PMI, or age and PMI by ANCOVA, the disease group differences for these 13 proteins remained significant (*P* < 0.05). With these corrections, no additional proteins reached statistical significance.

Stepwise logistic regression analysis of these measures identified a combination of the values for ErbB3, IL-2, and PF4 as having predictive value between control and PD cases (*P* = 0.0001). Analysis by ROC with all three factors showed sensitivity of 86.67% and specificity of 87.5%, and an AUC of 0.917. As expected, TH values alone had significant predictive value for discriminating control from PD cases (*P* = 0.0001) with ROC analysis showing sensitivity of 80%, specificity of 85.71%, and AUC 0.914. Including TH values with the 13 proteins in step-wise regression analysis produced a different outcome; CCL28 in combination with TH had significant predictive value (*P* < 0.0001), with sensitivity of 80%, specificity of 78.7%, and AUC of 0.938. Repeating the logistic regression analysis to determine if any of these measures gave predictive value between ILBD and PD cases identified PDGF-AA, with ROC analysis showing sensitivity of 72.2% and specificity of 62.5% and AUC of 0.722 (*P* = 0.015). Combining TH values with the 13 proteins showed IL-15 and TH values could discriminate between ILBD and PD (*P* = 0.0003), with sensitivity of 82.35 % and specificity of 85.71%, and AUC of 0.899. PDGF-AA levels alone gave discrimination between control and ILBD samples (*P* = 0.0014) with ROC analysis showing sensitivity of 66.67%, specificity of 77.78%, and AUC of 0.811. TH values alone discriminated between control and ILBD samples (*P* = 0.0157, sensitivity of 60%, specificity of 70.59%, and AUC of 0.737). Summary of these findings are shown in Table 4.

### Correlation analyses between measures

Multiple correlation analyses were carried out for proteins that showed significant One-way ANOVA differences to indicate if levels of any of these correlated with each other. In SN, IL-5, IL-15, and MIG levels positively correlated with each other, while IL-6sR did not correlate with any measure (Table 5). This suggests that in samples showing inflammatory activation, a network of similar factors become upregulated. Significant correlations between some of the striatum proteins were identified (Table 6). Proinflammatory cytokines in this table showed significant positive correlations with each other, while there was also significant positive correlation between the PDGF-AA and PDGF-BB. The negative correlations between PDGF-BB, and IFN-γ or TNF-α suggested that the levels of this growth factor were affected by inflammation; this was not observed for PDGF-AA (Table 6).

**Table 5:** **Correlation between significant protein measures and with TH in substantia nigra**.

	**IL-15**	**IL5**	**MIG**	**IL-6SR**
TH (R)	**0.315**	0.151	0.133	−0.075
(P)	**0.029**	0.306	0.367	0.61
IL-15		**0.737**	**0.488**	−0.191
		<**0.0001**	**0.0002**	0.166
IL-5			**0.656**	−0.254
			<**0.0001**	0.064
MIG				−0.177
				0.195

*(R) - Pearson correlation coefficient. (P) - P values. Values highlighted in bold indicate statistically significant correlation between measures*.

**Table 6:** **Significant Correlation between key protein measures in striatum**.

		Dtk	ErbB3			
CCL28	(R)	0.50	0.47			
	(P)	0.0002	0.0007			
		Dtk				
ErbB3	(R)	0.79				
	(P)	<0.0001				
		PDGF-AA	PF4			
HCC-1	(R)	0.46	0.37			
	(P)	0.0009	0.008			
		IL-2	IL-15	IFN-γ	PDGF-BB	TNF-α
IL-1ra	(R)	0.70	0.53	0.76	−0.385	0.566
	(P)	<0.0001	0.0001	<0.001	0.0063	<0.0001
		IL-15	IFN-γ	PDGF-BB	TNF-α	
IL-2	(R)	0.86	0.65	−0.45	0.80	
	(P)	<0.0001	<0.0001	0.0012	<0.0001	
		IFN-γ	PDGF-BB	TNF-α		
IL-15	(R)	0.56	0.42	0.74		
	(P)	0.0002	0.0026	<0.0001		
		PDGF-BB				
IFN-γ	(R)	−0.442				
	(P)	0.0015				
		PDGF-BB	PF4			
PDGF-AA	(R)	0.681	0.292			
	(P)	<0.0001	0.0418			
		PF4	IL-18 BPa	TNFa		
PDGF-BB	(R)	0.46	0.34	−0.35		
	(P)	0.0008	0.016	0.0128		
		TNFa				
IL-18BPa	(R)	−0.284				
	(P)	−0.048				

### Correlation analysis of all measured array features with TH

Multiple correlation analyses were carried out for all of the SN measures against TH levels. The significant correlations are shown in Table 7. Although CD14 and urokinase plasminogen activator receptor (uPAR), two classical markers of inflammation, did not show significant disease group differences, levels of the inflammation-associated markers negatively correlated with TH levels. The same analysis carried out for all striatal measures compared to striatum TH levels (Table 7) only showed significant positive correlations with activated leukocyte cell adhesion molecule (ALCAM-1), platelet-endothelial cell adhesion molecular (PECAM-1) and endoglin, all considered vascular adhesion proteins. The one common molecule from both brain regions was PECAM-1, which is involved in the integrity of the cerebral vasculature. To further assess inflammatory changes, we also measured levels of the astrocytic marker GFAP and the microglial markers IBA-1 and TLR-2 in samples by western blots. As had been shown (Figure 1D), the values between groups for GFAP in striatum were not statistically different (Figure 1D), but analysis of TH and GFAP across all disease groups did show significant negative correlation (*R* = −0.399, *P* = 0.0071; Figure 1D). Further separating these values into disease groups gave significant correlation between the control group GFAP values and TH (*R* = −0.551, *P* = 0.0219), but not between TH and GFAP levels for the ILBD or PD groups. There were no correlations between TH levels in SN and levels of IBA-1 or TLR-2 (data not shown).

**Table 7:** **Significant Correlations between protein measures and TH**.

**Substantia nigra**	**TH**	**Striatum**	**TH**
CD14 (R)	−0.306	ALCAM (R)	0.308
(P)	0.036	(P)	0.0375
CEACAM	0.461	Endoglin	0.401
	0.001		0.0057
EG-VEGF	0.674	PECAM-1	0.347
	<0.0001		0.0183
EGFR	0.521	GFAP	−0.528
	0.0001		0.0002
GDNF	0.313		
	0.030		
IL-13	0.43		
	0.0023		
IL-15	0.315		
	0.029		
Lipocalin-2	−0.366		
	0.011		
LYVE-1	−0.366		
	0.010		
PECAM-1	0.371		
	0.0094		
SCF-R	0.731		
	<0.0001		
TNF-α	0.411		
	0.0037		
uPAR	−0.299		
	0.039		

*R, Pearson R correlation coefficient. See supplemental Table 1 for abbreviations for proteins*.

### TLR-2 expression in SN

To follow up on the findings from a recent paper that examined tissue sections of SN from control, ILBD and PD cases and showed early increased expression of TLR-2 (Doorn et al., 2014), we carried out biochemical measurement of TLR-2 by western blot in our SN samples. Our analyses showed no significant differences between disease groups, but data obtained indicated TLR-2 levels had strong correlation with a number of the inflammatory markers measured (Table 8).

**Table 8:** **Significant correlations between key protein measures and TLR-2 in substantia nigra**.

	**TLR-2**
IBA-1 (R)	0.565
(P)	0.0003
CD14	0.434
	0.0045
CXCL16	0.38
	0.0142
HCC-1	0.568
	0.0001
HGF	0.422
	0.0059
IL-2	0.377
	0.0152
IL-5	0.372
	0.0166
MCP-1	0.531
	0.0004
TNF-RII	0.643
	<0.0001
TRAIL-R3	0.362
	0.0201

*R, Pearson R correlation coefficient. See Supplemental Table 1 for abbreviations for proteins*.

### Microglial activation profiles in SN and striatum

**Figure 4:**
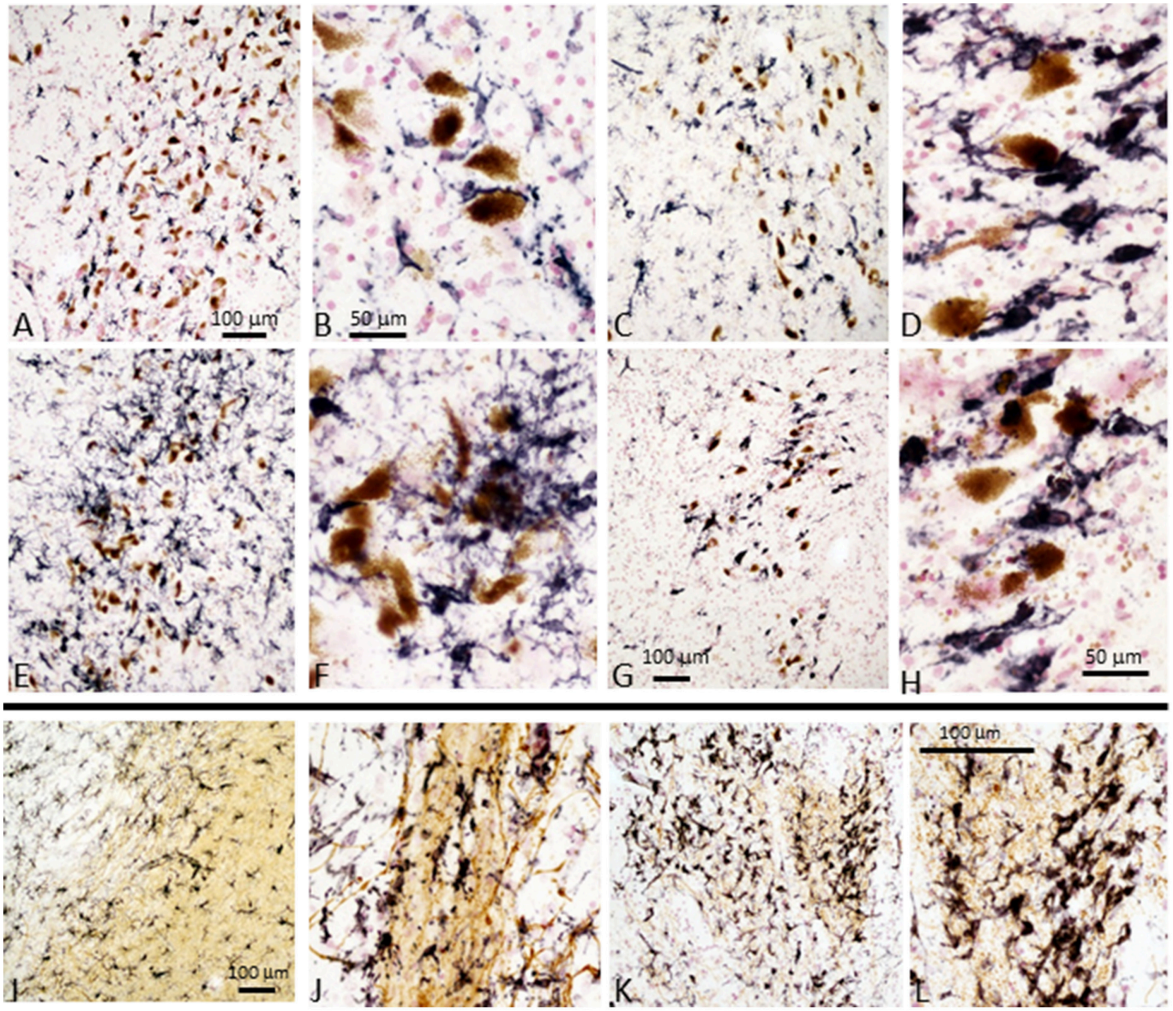
**Immunohistochemistry of microglia in tissue sections of SN and striatum**. **Substantia nigra:** Immunohistochemical staining of sections from Control **(A,B)**, ILBD **(C,D)**, and PD **(E,F)** and PD **(G,H)** from SN tissue sections. **(A,C,E,G)** are lower magnification of SN tissue sections showing progressive increase in intensity of HLA-DR immunoreactive microglia (purple) from **(A–E)**, but PD case **(G)** showed reduced microglia intensity (burnt-out pathology) consistent with loss of SN neurons. Higher magnification images **(B,D,F,H)** illustrate the changes in morphology of microglia with progressive activation with reduction in burnt-out PD case. **(H)** shows microglia associated free neuromelanin. The preferential association of activated microglia with neuromelanin-containing dopaminergic neuron is apparent in all sections, even those from control cases. **Striatum**: Immunohistochemical staining of sections of Control **(I,J)** and PD **(K,L)** from striatum (putamen) stained with antibody LN3 to HLA-DR (purple) and tyrosine hydroxylase (brown). Control case **(I,J)** show association of microglia with tyrosine hydroxylase positive fibers. Stronger HLA-DR immunoreactivity **(K)** and activated morphology is apparent in association with remaining TH positive fibers in PD case **(K,L)**. Representative staining of sections from cases included in the antibody array series and are representative of each group.

We stained tissue sections of SN and striatum from representative control, ILBD and PD cases to characterize activated microglia present in the disease groups. Staining was carried out using antibody LN3, which recognizes the MHCII protein HLA-DR, and antibody IBA-1, which recognizes all microglia. Results show increased immunoreactivity for HLA-DR in microglia associated with neuromelanin-containing neurons in the SN pars compacta, even in control cases (Figures 4A,B), with progressive change in microglial morphology in ILBD (Figures 4C,D), and PD cases (Figures 4E,F). The presence of smaller numbers of intensely-stained activated microglia in the control cases (Figures 4A,B) suggest ongoing inflammation even in the absence of disease. A noticeable feature was a difference within the PD groups, where some cases appeared to have a very strong inflammatory response in the vicinity of SN neurons (Figures 4E,F), while in others, there was a noticeable reduction in number of activated microglia (Figures 4G,H). Immunohistochemistry with antibody IBA-1 reveals greater numbers of microglia, but did not discriminate activated microglia in these sections (data not shown*)*. For striatum, the sections were double-stained with antibody to TH (brown) to reveal the location of positive fibers. In control striatum, there were TH positive fibers throughout the structure, while in the PD cases, most of the TH fibers were lost. In Figures 4J–L particularly intense microglial response was observed in the vicinity of surviving TH fibers. Again, HLA-DR-positive microglia were also seen in control striatum sections (Figures 4I,J), though with a less-activated morphology compared to those in PD cases (Figures 4K,L).

### Does the presence of AD-type pathology in brains affect inflammatory protein levels?

One limitation of human disease-focused studies can be the presence of mixed pathologies within the same tissue samples. For this study, the possible effects of age-associated AD-type pathology, namely plaques and tangles, on the inflammatory environment might be a confound factor. Although the primary diagnoses criteria used for case selection was the presence or absence of PD, along with the absence of AD, age-associated pathology was a feature of many of the cases used (Table 1). As the routine neuropathology diagnosis of each donor brains included an assessment of the overall burden of AD-type pathology by assessing the plaque and tangle scores of each brain, we used the above scores to identify any correlations of individual SN and striatum array measures with plaque and tangle scores using Spearman rank correlation analysis (Table 9). Proteins Dtk, ErBb3, IL-1ra, and IL-5 that had shown significant disease group differences in the antibody arrays for striatum showed significant correlations with tangle scores (Table 9, bold).

**Table 9:** **Correlations between Alzheimer's-type pathology and protein measures**.

**Plaques**			**Tangles**		
**Protein**	***R***	***P***	**Protein**	***R***	***P***
**SUBSTANTIA NIGRA**					
ALCAM	−0.335	0.012	Dtk	−0.276	0.041
CCL28	0.441	0.0008	PDGF-AA	−0.303	0.024
CD14	−0.381	0.004			
Endoglin	−0.416	0.002			
ErbB3	−0.323	0.016			
Flt-3L	−0.324	0.016			
IL-18Bpa	0.361	0.007			
PDGF-AA	−0.271	0.045			
PECAM-1	−0.343	0.010			
PF4	0.277	0.041			
uPAR	−0.299	0.027			
**STRIATUM**					
L-tactin	−0.352	0.013	AR	0.346	0.015
MCP-2	−0.34	0.017	**Dtk**	−**0.462**	**0.0008**
MIP-3a	−0.415	0.003	**ErbB3**	−**0.397**	**0.005**
OPN	−0.311	0.029	FGF-7	0.299	0.037
Trappin-2	−0.308	0.031	**IL-1ra**	**0.29**	**0.043**
uPAR	−0.317	0.026	**IL-5**	**0.295**	**0.039**
			IP-10	0.36	0.011
			NT-4	0.458	0.0009

*Plaques, Histological plaque score 0-15; Tangles, Histological tangle score 0-15; R, Spearman rank correlation. See Supplemental Table 1 for abbreviations for proteins. Results in bold identify proteins that showed significant differences between control, ILBD, and PD groups*.

## Discussion

This work is the first description of large-scale unbiased multiplex quantitative protein profiling of tissue from human SN and striatum to elucidate changes in inflammatory and related molecules that might be involved in PD pathology, though this platform was used to screen sera from patients with Parkinsonian syndromes for inflammatory protein changes (Mahlknecht et al., 2012). We measured the levels of 158 different proteins in SN and striatum samples from control, ILBD, and PD cases. The study was based on the hypothesis that inflammatory molecules that become upregulated in ILBD cases might be more likely involved in disease mechanisms. ILBD seems to represent a precursor stage to PD; these subjects have no diagnosed movement disorders or dementia, but certain amounts of LB pathology, loss of dopaminergic neurons and reduced levels of TH (Beach et al., 2008; Caviness et al., 2011; Iacono et al., 2015). In agreement with our earlier studies, we showed this group of tissue samples had intermediate TH values between control and PD cases in striatum (Beach et al., 2008, 2009).

The major findings from this study were the identification of candidate inflammatory or growth factor molecules that showed significant disease group differences. We identified four proteins in SN, and 13 proteins in striatum that had One-way ANOVA differences, and *post-hoc* differences between disease groups with *P* < 0.05, though these statistical analysis did not take account for multiple comparisons. The other feature of the study was the identification of a different inflammatory signature for SN compared to striatum. The only molecule that changed significantly for both brain regions was IL-5. Independent validation of these findings are now required using sensitive methodologies in a new group of tissue samples.

An inherent limitation to the approach using human tissues was the highly variable nature of disease and inflammatory changes within each group. This large variability for many measures reduced the potential of detecting disease group differences. As demonstrated in Figure 1, there was considerable variability in the values for TH as the fundamental biochemical measure of disease severity. This could also be seen in most individual measures (see scatter plots in Figures 2, 3 as examples) particularly within the control groups. Related to this was sensitivity of detection for certain proteins. It was observed that a number of samples within each group had values below the LOD of the assays. Although this was observed for IL-5, IL-15, and MIG in SN, and IL-2, TNF-α, IL1-RA, IL-15, and IFN-γ in striatum (Figures 2, 3), a clear increase in the number of PD samples having higher positive values was apparent, confirming the changes in these cytokines in the diseased samples.

The analyses of protein levels showed a higher percentage of proteins being below the LOD in SN compared to striatum. Of the 158 array proteins being measured, 101 of these proteins could be detected and measurable in striatum, while 81 were measurable in SN. The difference in protein profiles between SN and striatum did not appear to be due to different sensitivity between tissues. All of the proteins that showed significant differences between disease groups in striatum samples could be detected in SN, while the reverse situation was present for significant SN proteins in striatum (Table 3). This feature provided support for the observation of differences in inflammatory features between SN and striatum.

Fundamental to this study was also a demonstration of differences in appearance of microglia in disease-affected tissues. Immunohistochemical staining of selected tissue sections of SN and striatum for the presence of morphologically activated microglia using antibody LN3 identified a progressive change in appearance of microglia associated with increasing pathology. This was particularly apparent in SN; however, two patterns of activated microglia could be seen in PD cases. Some cases had large numbers of highly-activated microglia potentially indicative of an active pathological process; while others showed a reduced number of microglia with many having intracellular neuromelanin potentially indicative of “burnt-out” pathology (Figures 4G,H compared to Figures 4E,F). Similar patterns were apparent in striatum in association with TH positive fibers. One noticeable feature was the presence of considerable numbers of activated microglia in SN and striatum from control cases that had absence of LB pathology.

There were two patterns of changes in proteins between disease groups. For those proteins that had reduced levels with progression to PD, this could indicate loss of “protective” functions as a disease feature. For those proteins that showed increased levels with progression to PD, this indicated a possible role in enhancing inflammatory pathology. In SN, there were increases in PD for cytokines IL-5, IL-15, and MIG, but decrease in levels of IL-6sR. In striatum, there were decreases in PD compared to control for PDGF-AA, PDGF-BB, ErbB3, Dtk, PF4 and HCC-1, IL-18BPa and CCL28, but increases in PD for cytokines IL-2, IFN-γ, TNF-α, IL-1Ra, and IL-15. The factors downregulated are either inflammatory antagonists or growth factors, while the factors upregulated are inflammation-associated cytokines. With the exception of IL-2, TNF-α, and IFN-γ, the other factors we identified have not been associated with PD in previous animal or human studies.

Of the highlighted proteins, some features as they relate to possible brain function will be discussed. IL-5 has been described as a Th2-cell cytokine whose expression is upregulated in inflammatory-associated conditions associated with parasitic infections, similar to IL-4 or IL-13, with secretion of IL-5 by astrocytes and microglia having been described (Sawada et al., 1993). IL-5 can also be a colony stimulating factor for macrophages and microglia (Ringheim, 1995; Liva and de Vellis, 2001). By comparison, IL-15 expression in diseased brain has been more widely studied than IL-5. Both neurotoxic and proinflammatory consequences of IL-15 have been observed in brain. IL-15 receptors are present on neurons, and treatment of neurons *in vivo* with IL-15 resulted in reduced neurite outgrowth and reduction in expression of microtubule-associated brain (MAP)-2 (Huang et al., 2009). IL-15 can enhance the cytotoxic potential of CD4+ T cells in multiple sclerosis (Broux et al., 2015). IL-15 also has an effect at the blood-brain barrier resulting in weakening of tight junctions and increased permeability (Stone et al., 2011). Increased expression of MIG by microglia in brain has been demonstrated in response to IFN-γ and also IL-9 (Carter et al., 2007; Ellis et al., 2010; Ding et al., 2015). MIG activates cellular signaling through the receptor CXCR3, which is shared with CXCL10 (interferon gamma induced protein 10–IP-10). Compared to MIG, IP10 was not consistently detected in SN samples. Interpreting the decrease in levels of IL-6sR in PD compared to ILBD samples is complex as IL-6sR functions can have both agonist and antagonist properties. IL-6 interaction with IL-6sR interaction enhances signaling on cells expressing the other IL-6 receptor gp130, while blocking signaling on cells expressing both IL-6R and gp130. Microglia express IL-6R as well as gp130 so changes in IL-6sR levels might block rather than enhance activation of microglia. However, other studies have shown that IL-6 signaling in brain is predominantly enhanced by IL-6sR (Burton et al., 2011, 2013; Campbell et al., 2014).

The different pattern of changes for proteins were observed in striatum compared to SN was suggestive of a decline in growth factors along with enhanced levels of different inflammatory proteins were ongoing. Both PDGF-AA and PDGF-BB levels showed disease differences in striatum. It has been known that PDGF can be neurotrophic for transplanted dopaminergic neurons and induces neurogenesis (Smits et al., 1993; Mohapel et al., 2005). Clinical trials using intraventricular infusion of PDGF-BB in PD patients as a therapeutic treatment to support dopaminergic neurons are ongoing (Paul et al., 2015). In Table 5, the significant negative correlations of PDGF-BB levels with those of inflammatory markers IL-1RA, IL-2, IL-15, IFN-γ, and TNF-α suggested that levels were negatively affected by degree of inflammatory activation. The correlation between PDGF-BB and TH levels in striatum was close to significance (*P* = 0.052). CCL-28 is expressed by epithelial cells and has chemotactic properties for certain immune cells. Increased expression in periphery has been associated with inflammation, however, in brain, it has only been identified in neurons rather than glial cells (Liu et al., 2012). In a model of epilepsy, downregulated expression of CCL28 was associated with loss of neurons (Liu et al., 2012). Dtk (receptor tyrosine kinase TYRO3) is also predominantly expressed by neurons (Prieto et al., 2007; Zhao et al., 2012). Its function in the PD brain is unclear, but overexpression of Dtk in an AD mouse model resulted in reduced production of amyloid beta (Aβ) peptide (Zheng et al., 2012). Interleukin-18 binding protein α (IL-18BPa) is high-affinity antagonist for the proinflammatory cytokine IL-18 (interferon gamma inducing factor). Both factors normally occur in balance, but a decrease in IL-18BPa can be accompanied with increased IL-18 (Dinarello et al., 2013). This factor has not been studied in human brain Platelet factor 4 (PF4), also known as CXCL4, is a chemokine involved in wound repair and inflammation. It has anti-angiogenic properties, and is an antagonist for the basic fibroblast growth factor receptor (Sulpice et al., 2002; Chadderton and Stringer, 2003). ErbB3 is an epidermal growth factor-related receptor tyrosine kinase with neuregulin as a ligand. ErbB3 is widely expressed in brain in gray matter, white matter, neurons, astrocytes, endothelial cells, and oligodendrocytes (Ozaki et al., 1998; Steiner et al., 1999; Calaora et al., 2001; Lok et al., 2009; Sharif et al., 2009). Examination of human PD striatum samples for EGF and ErbB receptors 1-4 demonstrated different results from this study (Iwakura et al., 2005). A decrease of EGF in PD samples was reported, along with a decrease in ErbB1 and ErbB2, but not ErbB3 (Iwakura et al., 2005). We did not identify differences in EGF levels in striatum or SN samples in this study. HCC-1 (CCL-14) has not been studied in brain, but has been characterized as a marker for alternatively activated/anti-inflammatory macrophages (Jaguin et al., 2013). In the striatum samples, there was a significant increase between ILBD and PD samples.

Five of the inflammatory cytokines associated with disease differences were progressively upregulated in striatum between control, ILBD, and PD samples. IFN-γ is a potent proinflammatory cytokine primarily produced by T lymphocytes and natural killer cells rather than by brain resident cells. IFN-γ has been shown to have a central role in mediating dopaminergic cell loss or deterioration of the nigrostriatal pathway in PD animal models (Mount et al., 2007; Brochard et al., 2009; Chakrabarty et al., 2011; Barcia et al., 2012; Mangano et al., 2012). By contrast, IL-1RA is protective and blocks the binding of the cytokines IL-1α or IL-1β to the IL-1 receptor. Its increased levels are suggestive of inflammation due to elevated levels of IL-1α or IL-1β. This cannot be confirmed in this study as IL-1α and IL-1β were not detectable in most SN or striatum samples. IL-1RA has significant neuroprotective properties in situations with brain injury with increased expression by neurons in AD and Pick's disease brains (Yasuhara et al., 1997; Sanderson et al., 1999; Yang et al., 1999; Vecil et al., 2000; Masada et al., 2003). IL-2 is also a T-lymphocyte produced cytokine that is involved in cellular immune responses mediated by T-lymphocytes. Increased IL-2 levels were reported in an earlier study in PD striatum (Mogi et al., 1996). TNF-α is been the cytokine most widely implicated in the pathogenesis of PD. The majority of studies have focused on TNF-α in various animal models not in human brain (Nagatsu et al., 2000a; Gayle et al., 2002; Carvey et al., 2005a; Zhao et al., 2007; De Lella Ezcurra et al., 2010; Chertoff et al., 2011; Daniele et al., 2015). Sensitivity of detection of this cytokine was an issue due to the number of samples with negative values included in the analysis. However, comparing TNF-α levels in SN control samples with control striatum samples illustrate difference between these two brain regions. In SN, half of the control samples had detectable levels of TNF-α, while the striatal control samples were all below the level of detection. As can be seen, the distribution of values in SN between control and PD were not significantly different (*P* = 0.144), while in striatum there was an increasing number of positive samples in the ILBD and PD groups (*P* = 0.008). Although the values were low, the amount of TNF-α detected in SN was overall higher than in striatum. Mean values in PD SN samples were 10.1 pg/ml compared to 3.5 pg/ml in striatum PD samples.

Although interpreting the implications of these findings for identifying therapeutic targets needs to be done with caution, interactions of different factors and also the presence of other pathologies needs to be considered. Correlation of SN measures with TH levels identified one panel of 13 proteins, while a panel of four proteins correlated with TH levels in striatum. One common feature was the number of vascular markers—this suggested that changes in vascular integrity could be a feature of PD pathogenesis. Pathology-based studies have shown increased vascular degeneration in PD affected brain tissue, as well as aberrant angiogenesis that also can promote vascular degeneration (Desai et al., 2009; Guan et al., 2013). The possible confounding effect of AD-type pathology also needs to be considered when interpreting findings. It has been recently described that many subjects diagnosed clinically with PD show evidence of additional neuropathology upon postmortem examination (Dugger et al., 2014). As demonstrated in Table 9, there were significant correlations between a number of these inflammatory proteins with plaque and tangle pathology, even though the AD pathology measures used in our analyses did not directly assess plaques and tangles in SN and striatum but were used as an overall index of brain load of AD-type pathology.

In summary, we had defined different inflammatory profiles for SN and striatum using an antibody array assay that measured 158 different biologically active proteins in tissue from control, ILBD, and PD cases. The study has identified candidate molecules whose role(s) in accelerating PD pathology can be validated in further analyses. One of the problems highlighted in this study is the heterogeneity in disease or aging changes in these carefully selected cases, as well as the presence of other neuropathologies that can affect measures. Variability is inevitable in human samples, but potentially masked some of the disease group differences. The very large range of values found for each of the factors suggests other causes beside PD pathology might be affecting levels of molecules in tissue. The use of correlation analyses along with assessment of disease group differences provided interesting leads to follow up. The strength of this application is the quality of the postmortem tissue used. These were collected and stored under defined conditions with short postmortem delays so the stability of these measured molecules should be assured (Beach et al., 2015). Further studies though will require methodology with higher sensitivity to be able to measure the low levels of key cytokines in all tissue samples.

## Author contributions

DW planned the study, coordinated sample processing and analysis, performed data analysis, manuscript writing, and coordinating with funding agent. LL assisted in these processes. GS and LS selected and dissected the tissue samples used in the study and provided neuropathology consultations. JC and CA provided clinical assessments premortem on many of the recruited subjects in the study. TB provided neuropathology diagnosis, assisted in sample selection and coordinated tissue collection.

### Conflict of interest statement

The authors declare that the research was conducted in the absence of any commercial or financial relationships that could be construed as a potential conflict of interest.

## References

[B1] Aarsland D. Zaccai J. Brayne C. (2005). A systematic review of prevalence studies of dementia in Parkinson's disease. Mov. Disord. 20, 1255–1263. 10.1002/mds.20527 16041803

[B2] Acosta S. A. Tajiri N. de la Pena I. Bastawrous M. Sanberg P. R. Kaneko Y.. (2015). Alpha-synuclein as a pathological link between chronic traumatic brain injury and Parkinson's disease. J. Cell. Physiol. 230, 1024–1032. 10.1002/jcp.24830 25251017 PMC4328145

[B3] Ancolio K. Alves da Costa C. Uéda K. Checler F. (2000). Alpha-synuclein and the Parkinson's disease-related mutant Ala53Thr-alpha-synuclein do not undergo proteasomal degradation in HEK293 and neuronal cells. Neurosci. Lett. 285, 79–82. 10.1016/S0304-3940(00)01049-1 10793231

[B4] Baba M. Nakajo S. Tu P. H. Tomita T. Nakaya K. Lee V. M.. (1998). Aggregation of alpha-synuclein in Lewy bodies of sporadic Parkinson's disease and dementia with Lewy bodies. Am. J. Pathol. 152, 879–884. 9546347 PMC1858234

[B5] Barcia C. Ros C. M. Annese V. Gómez A. Ros-Bernal F. Aguado-Llera D.. (2012). IFN-γ signaling, with the synergistic contribution of TNF-α, mediates cell specific microglial and astroglial activation in experimental models of Parkinson's disease. Cell Death Dis. 3, e379. 10.1038/cddis.2012.123 22914327 PMC3434670

[B6] Beach T. G. Adler C. H. Lue L. Sue L. I. Bachalakuri J. Henry-Watson J.. (2009). Unified staging system for Lewy body disorders: correlation with nigrostriatal degeneration, cognitive impairment and motor dysfunction. Acta Neuropathol. 117, 613–634. 10.1007/s00401-009-0538-8 19399512 PMC2757320

[B7] Beach T. G. Adler C. H. Sue L. I. Peirce J. B. Bachalakuri J. Dalsing-Hernandez J. E.. (2008). Reduced striatal tyrosine hydroxylase in incidental Lewy body disease. Acta Neuropathol. 115, 445–451. 10.1007/s00401-007-0313-7 17985144 PMC2724592

[B8] Beach T. G. Adler C. H. Sue L. I. Serrano G. Shill H. A. Walker D. G.. (2015). Arizona study of aging and neurodegenerative disorders and brain and body donation program. Neuropathology 35, 354–389. 10.1111/neup.12189 25619230 PMC4593391

[B9] Beach T. G. Sue L. I. Walker D. G. Sabbagh M. N. Serrano G. Dugger B. N.. (2012). Striatal amyloid plaque density predicts Braak neurofibrillary stage and clinicopathological Alzheimer's disease: implications for amyloid imaging. J. Alzheimers. Dis. 28, 869–876. 10.3233/JAD-2011-111340 22112552 PMC3760731

[B10] Béraud D. Hathaway H. A. Trecki J. Chasovskikh S. Johnson D. A. Johnson J. A.. (2013). Microglial activation and antioxidant responses induced by the Parkinson's disease protein α-synuclein. J. Neuroimmune Pharmacol. 8, 94–117. 10.1007/s11481-012-9401-0 23054368 PMC3582877

[B11] Boka G. Anglade P. Wallach D. Javoy-Agid F. Agid Y. Hirsch E. C. (1994). Immunocytochemical analysis of tumor necrosis factor and its receptors in Parkinson's disease. Neurosci. Lett. 172, 151–154. 10.1016/0304-3940(94)90684-X 8084523

[B12] Botta-Orfila T. Tolosa E. Gelpi E. Sànchez-Pla A. Martí M.-J. Valldeoriola F.. (2012). Microarray expression analysis in idiopathic and LRRK2-associated Parkinson's disease. Neurobiol. Dis. 45, 462–468. 10.1016/j.nbd.2011.08.033 21946334

[B13] Brochard V. Combadière B. Prigent A. Laouar Y. Perrin A. Beray-Berthat V.. (2009). Infiltration of CD4+ lymphocytes into the brain contributes to neurodegeneration in a mouse model of Parkinson disease. *J. Clin*. Invest. 119, 182–192. 10.1172/JCI36470 19104149 PMC2613467

[B14] Broux B. Mizee M. R. Vanheusden M. van der Pol S. van Horssen J. Van Wijmeersch B.. (2015). IL-15 amplifies the pathogenic properties of CD4+CD28- T cells in multiple sclerosis. J. Immunol. 194, 2099–2109. 10.4049/jimmunol.1401547 25617471

[B15] Burton M. D. Rytych J. L. Freund G. G. Johnson R. W. (2013). Central inhibition of interleukin-6 trans-signaling during peripheral infection reduced neuroinflammation and sickness in aged mice. Brain. Behav. Immun. 30, 66–72. 10.1016/j.bbi.2013.01.002 23354002 PMC3641158

[B16] Burton M. D. Sparkman N. L. Johnson R. W. (2011). Inhibition of interleukin-6 trans-signaling in the brain facilitates recovery from lipopolysaccharide-induced sickness behavior. J. Neuroinflammation 8:54. 10.1186/1742-2094-8-54 21595956 PMC3113341

[B17] Calaora V. Rogister B. Bismuth K. Murray K. Brandt H. Leprince P.. (2001). Neuregulin signaling regulates neural precursor growth and the generation of oligodendrocytes *in vitro*. J. Neurosci. 21, 4740–4751. 11425901 10.1523/JNEUROSCI.21-13-04740.2001 PMC6762347

[B18] Campbell I. L. Erta M. Lim S. L. Frausto R. May U. Rose-John S.. (2014). Trans-signaling is a dominant mechanism for the pathogenic actions of interleukin-6 in the brain. J. Neurosci. 34, 2503–2513. 10.1523/JNEUROSCI.2830-13.2014 24523541 PMC6802757

[B19] Carter S. L. Müller M. Manders P. M. Campbell I. L. (2007). Induction of the genes for Cxcl9 and Cxcl10 is dependent on IFN-gamma but shows differential cellular expression in experimental autoimmune encephalomyelitis and by astrocytes and microglia *in vitro*. Glia 55, 1728–1739. 10.1002/glia.20587 17902170

[B20] Carvey P. M. Chen E. Y. Lipton J. W. Tong C. W. Chang Q. A. Ling Z. D. (2005a). Intra-parenchymal injection of tumor necrosis factor-alpha and interleukin 1-beta produces dopamine neuron loss in the rat. J. Neural Transm. 112, 601–612. 10.1007/s00702-004-0222-z 15583962

[B21] Carvey P. M. Punati A. Newman M. B. (2006). Progressive dopamine neuron loss in Parkinson's disease: the multiple hit hypothesis. Cell Transplant. 15, 239–250. 10.3727/000000006783981990 16719059

[B22] Carvey P. M. Zhao C. H. Hendey B. Lum H. Trachtenberg J. Desai B. S.. (2005b). 6-Hydroxydopamine-induced alterations in blood-brain barrier permeability. Eur. J. Neurosci. 22, 1158–1168. 10.1111/j.1460-9568.2005.04281.x 16176358

[B23] Caviness J. N. Adler C. H. Hentz J. G. Shill H. A. Evidente V. G. H. Driver-Dunckley E. D.. (2011). Incidental Lewy body disease: electrophysiological findings suggesting pre-clinical Lewy body disorders. Clin. Neurophysiol. 122, 2426–2432. 10.1016/j.clinph.2011.03.033 21616709 PMC3164932

[B24] Chadderton N. S. Stringer S. E. (2003). Interaction of platelet factor 4 with fibroblast growth factor 2 is stabilised by heparan sulphate. Int. J. Biochem. Cell Biol. 35, 1052–1055. 10.1016/S1357-2725(02)00299-6 12672474

[B25] Chakrabarty P. Ceballos-Diaz C. Lin W.-L. Beccard A. Jansen-West K. McFarland N. R.. (2011). Interferon-γ induces progressive nigrostriatal degeneration and basal ganglia calcification. Nat. Neurosci. 14, 694–696. 10.1038/nn.2829 21572432 PMC3780582

[B26] Chertoff M. Di Paolo N. Schoeneberg A. Depino A. Ferrari C. Wurst W.. (2011). Neuroprotective and neurodegenerative effects of the chronic expression of tumor necrosis factor α in the nigrostriatal dopaminergic circuit of adult mice. Exp. Neurol. 227, 237–251. 10.1016/j.expneurol.2010.11.010 21093436

[B27] Choi D.-Y. Liu M. Hunter R. L. Cass W. A. Pandya J. D. Sullivan P. G.. (2009). Striatal neuroinflammation promotes Parkinsonism in rats. PLoS ONE 4:e5482. 10.1371/journal.pone.0005482 19424495 PMC2674956

[B28] Codolo G. Plotegher N. Pozzobon T. Brucale M. Tessari I. Bubacco L.. (2013). Triggering of inflammasome by aggregated α-synuclein, an inflammatory response in synucleinopathies. PLoS ONE 8:e55375. 10.1371/journal.pone.0055375 23383169 PMC3561263

[B29] Conway K. A. Harper J. D. Lansbury P. T. (1998). Accelerated *in vitro* fibril formation by a mutant alpha-synuclein linked to early-onset Parkinson disease. Nat. Med. 4, 1318–1320. 10.1038/3311 9809558

[B30] Couch Y. Alvarez-Erviti L. Sibson N. R. Wood M. J. A. Anthony D. C. (2011). The acute inflammatory response to intranigral α-synuclein differs significantly from intranigral lipopolysaccharide and is exacerbated by peripheral inflammation. J. Neuroinflammation 8:166. 10.1186/1742-2094-8-166 22122884 PMC3239418

[B31] Daniele S. G. Béraud D. Davenport C. Cheng K. Yin H. Maguire-Zeiss K. A. (2015). Activation of MyD88-dependent TLR1/2 signaling by misfolded α-synuclein, a protein linked to neurodegenerative disorders. Sci. Signal. 8, ra45. 10.1126/scisignal.2005965 25969543 PMC4601639

[B32] De Lella Ezcurra A. L. Chertoff M. Ferrari C. Graciarena M. Pitossi F. (2010). Chronic expression of low levels of tumor necrosis factor-alpha in the substantia nigra elicits progressive neurodegeneration, delayed motor symptoms and microglia/macrophage activation. Neurobiol. Dis. 37, 630–640. 10.1016/j.nbd.2009.11.018 19969084

[B33] Desai B. S. Monahan A. J. Carvey P. M. Hendey B. (2007). Blood-brain barrier pathology in Alzheimer's and Parkinson's disease: implications for drug therapy. Cell Transplant. 16, 285–299. 10.3727/000000007783464731 17503739

[B34] Desai B. S. Schneider J. A. Li J.-L. Carvey P. M. Hendey B. (2009). Evidence of angiogenic vessels in Alzheimer's disease. J. Neural Transm. 116, 587–597. 10.1007/s00702-009-0226-9 19370387 PMC2753398

[B35] Dinarello C. A. Novick D. Kim S. Kaplanski G. (2013). Interleukin-18 and IL-18 binding protein. Front. Immunol. 4:289. 10.3389/fimmu.2013.00289 24115947 PMC3792554

[B36] Ding X. Cao F. Cui L. Ciric B. Zhang G.-X. Rostami A. (2015). IL-9 signaling affects central nervous system resident cells during inflammatory stimuli. Exp. Mol. Pathol. 99, 570–574. 10.1016/j.yexmp.2015.07.010 26216406 PMC6524950

[B37] Doorn K. J. Moors T. Drukarch B. van de Berg W. D. Lucassen P. J. van Dam A.-M. (2014). Microglial phenotypes and toll-like receptor 2 in the substantia nigra and hippocampus of incidental Lewy body disease cases and Parkinson's disease patients. Acta Neuropathol. Commun. 2, 90. 10.1186/s40478-014-0090-1 25099483 PMC4224021

[B38] Dugger B. N. Adler C. H. Shill H. A. Caviness J. Jacobson S. Driver-Dunckley E.. (2014). Concomitant pathologies among a spectrum of parkinsonian disorders. Parkinsonism Relat. Disord. 20, 525–529. 10.1016/j.parkreldis.2014.02.012 24637124 PMC4028418

[B39] Duke D. C. Moran L. B. Kalaitzakis M. E. Deprez M. Dexter D. T. Pearce R. K. B.. (2006). Transcriptome analysis reveals link between proteasomal and mitochondrial pathways in Parkinson's disease. Neurogenetics 7, 139–148. 10.1007/s10048-006-0033-5 16699787

[B40] Durrenberger P. F. Grünblatt E. Fernando F. S. Monoranu C. M. Evans J. Riederer P.. (2012). Inflammatory pathways in Parkinson's Disease; a BNE microarray study. Parkinsons Dis. 2012:214714. 10.1155/2012/214714 22548201 PMC3324922

[B41] Ellis S. L. Gysbers V. Manders P. M. Li W. Hofer M. J. Müller M.. (2010). The cell-specific induction of CXC chemokine ligand 9 mediated by IFN-gamma in microglia of the central nervous system is determined by the myeloid transcription factor PU.1. J. Immunol. 185, 1864–1877. 10.4049/jimmunol.1000900 20585034 PMC2925661

[B42] Elstner M. Morris C. M. Heim K. Lichtner P. Bender A. Mehta D.. (2009). Single-cell expression profiling of dopaminergic neurons combined with association analysis identifies pyridoxal kinase as Parkinson's disease gene. Ann. Neurol. 66, 792–798. 10.1002/ana.21780 20035503 PMC4034432

[B43] Fellner L. Irschick R. Schanda K. Reindl M. Klimaschewski L. Poewe W.. (2013). Toll-like receptor 4 is required for α-synuclein dependent activation of microglia and astroglia. Glia 61, 349–360. 10.1002/glia.22437 23108585 PMC3568908

[B44] Fischer R. Maier O. (2015). Interrelation of oxidative stress and inflammation in neurodegenerative disease: role of TNF. Oxid. Med. Cell. Longev. 2015:610813. 10.1155/2015/610813 25834699 PMC4365363

[B45] Fujiwara H. Hasegawa M. Dohmae N. Kawashima A. Masliah E. Goldberg M. S.. (2002). alpha-Synuclein is phosphorylated in synucleinopathy lesions. Nat. Cell Biol. 4, 160–164. 10.1038/ncb748 11813001

[B46] Gao H.-M. Zhang F. Zhou H. Kam W. Wilson B. Hong J.-S. (2011). Neuroinflammation and α-synuclein dysfunction potentiate each other, driving chronic progression of neurodegeneration in a mouse model of Parkinson's disease. Environ. Health Perspect. 119, 807–814. 10.1289/ehp.1003013 21245015 PMC3114815

[B47] Gardai S. J. Mao W. Schüle B. Babcock M. Schoebel S. Lorenzana C.. (2013). Elevated alpha-synuclein impairs innate immune cell function and provides a potential peripheral biomarker for Parkinson's disease. PLoS ONE 8:e71634. 10.1371/journal.pone.0071634 24058406 PMC3751933

[B48] Gayle D. A. Ling Z. Tong C. Landers T. Lipton J. W. Carvey P. M. (2002). Lipopolysaccharide (LPS)-induced dopamine cell loss in culture: roles of tumor necrosis factor-alpha, interleukin-1beta, and nitric oxide. Brain Res. Dev. Brain Res. 133, 27–35. 10.1016/S0165-3806(01)00315-7 11850061

[B49] Ghosh D. Mondal M. Mohite G. M. Singh P. K. Ranjan P. Anoop A.. (2013). The Parkinson's disease-associated H50Q mutation accelerates α-Synuclein aggregation *in vitro*. Biochemistry 52, 6925–6927. 10.1021/bi400999d 24047453

[B50] Giráldez-Pérez R. Antolín-Vallespín M. Muñoz M. Sánchez-Capelo A. (2014). Models of α-synuclein aggregation in Parkinson's disease. Acta Neuropathol. Commun. 2, 176. 10.1186/s40478-014-0176-9 25497491 PMC4272812

[B51] Grünblatt E. Mandel S. Jacob-Hirsch J. Zeligson S. Amariglo N. Rechavi G.. (2004). Gene expression profiling of parkinsonian substantia nigra pars compacta; alterations in ubiquitin-proteasome, heat shock protein, iron and oxidative stress regulated proteins, cell adhesion/cellular matrix and vesicle trafficking genes. J. Neural Transm. 111, 1543–1573. 10.1007/s00702-004-0212-1 15455214

[B52] Gründemann J. Schlaudraff F. Liss B. (2011). UV-laser microdissection and mRNA expression analysis of individual neurons from postmortem Parkinson's disease brains. Methods Mol. Biol. 755, 363–374. 10.1007/978-1-61779-163-5_30 21761319

[B53] Guan J. Pavlovic D. Dalkie N. Waldvogel H. J. O'Carroll S. J. Green C. R.. (2013). Vascular degeneration in Parkinson's disease. Brain Pathol. 23, 154–164. 10.1111/j.1750-3639.2012.00628.x 22897695 PMC8029297

[B54] Harms A. S. Cao S. Rowse A. L. Thome A. D. Li X. Mangieri L. R.. (2013). MHCII is required for α-synuclein-induced activation of microglia, CD4 T cell proliferation, and dopaminergic neurodegeneration. J. Neurosci. 33, 9592–9600. 10.1523/JNEUROSCI.5610-12.2013 23739956 PMC3903980

[B55] Hauser M. A. Li Y.-J. Xu H. Noureddine M. A. Shao Y. S. Gullans S. R.. (2005). Expression profiling of substantia nigra in Parkinson disease, progressive supranuclear palsy, and frontotemporal dementia with parkinsonism. Arch. Neurol. 62, 917–921. 10.1001/archneur.62.6.917 15956162

[B56] Hirsch E. C. (1993). Does oxidative stress participate in nerve cell death in Parkinson's disease? Eur. Neurol. 33(Suppl. 1), 52–59. 8375433 10.1159/000118538

[B57] Huang Y.-S. Cheng S.-N. Chueh S.-H. Tsai Y.-L. Liou N.-H. Guo Y.-W.. (2009). Effects of interleukin-15 on neuronal differentiation of neural stem cells. Brain Res. 1304, 38–48. 10.1016/j.brainres.2009.09.009 19747902

[B58] Hunot S. Dugas N. Faucheux B. Hartmann A. Tardieu M. Debré P.. (1999). FcepsilonRII/CD23 is expressed in Parkinson's disease and induces, *in vitro*, production of nitric oxide and tumor necrosis factor-alpha in glial cells. J. Neurosci. 19, 3440–3447. 10212304 10.1523/JNEUROSCI.19-09-03440.1999 PMC6782235

[B59] Iacono D. Geraci-Erck M. Rabin M. L. Adler C. H. Serrano G. Beach T. G.. (2015). Parkinson disease and incidental Lewy body disease: just a question of time? Neurology 85, 1670–1679. 10.1212/WNL.0000000000002102 26468408 PMC4653112

[B60] Iwakura Y. Piao Y.-S. Mizuno M. Takei N. Kakita A. Takahashi H.. (2005). Influences of dopaminergic lesion on epidermal growth factor-ErbB signals in Parkinson's disease and its model: neurotrophic implication in nigrostriatal neurons. J. Neurochem. 93, 974–983. 10.1111/j.1471-4159.2005.03073.x 15857400

[B61] Jaguin M. Houlbert N. Fardel O. Lecureur V. (2013). Polarization profiles of human M-CSF-generated macrophages and comparison of M1-markers in classically activated macrophages from GM-CSF and M-CSF origin. Cell. Immunol. 281, 51–61. 10.1016/j.cellimm.2013.01.010 23454681

[B62] Janelidze S. Lindqvis D. Francardo V. Hall S. Zetterberg H. Blennow K. (2015). Increased CSF biomarkers of angiogenesis in Parkinson disease. Neurology 24, 1834–1842. 10.1212/WNL.0000000000002151 PMC4662706 26511451

[B63] Jiang T. Hoekstra J. Heng X. Kang W. Ding J. Liu J.. (2015). P2X7 receptor is critical in α-synuclein–mediated microglial NADPH oxidase activation. Neurobiol. Aging 36, 2304–2318. 10.1016/j.neurobiolaging.2015.03.015 25983062

[B64] Kim C. Ho D.-H. Suk J.-E. You S. Michael S. Kang J.. (2013). Neuron-released oligomeric α-synuclein is an endogenous agonist of TLR2 for paracrine activation of microglia. Nat. Commun. 4, 1562. 10.1038/ncomms2534 23463005 PMC4089961

[B65] Koprich J. B. Johnston T. H. Reyes M. G. Sun X. Brotchie J. M. (2010). Expression of human A53T alpha-synuclein in the rat substantia nigra using a novel AAV1/2 vector produces a rapidly evolving pathology with protein aggregation, dystrophic neurite architecture and nigrostriatal degeneration with potential to model the pat. Mol. Neurodegener. 5:43. 10.1186/1750-1326-5-43 21029459 PMC2984491

[B66] Lawand N. B. Saadé N. E. El-Agnaf O. M. Safieh-Garabedian B. (2015). Targeting α-synuclein as a therapeutic strategy for Parkinson's disease. Expert Opin. Ther. Targets 19, 1351–1360. 10.1517/14728222.2015.1062877 26135549

[B67] Licker V. Côte M. Lobrinus J. A. Rodrigo N. Kövari E. Hochstrasser D. F.. (2012). Proteomic profiling of the substantia nigra demonstrates CNDP2 overexpression in Parkinson's disease. J. Proteomics 75, 4656–4667. 10.1016/j.jprot.2012.02.032 22410244

[B68] Liu J. X. Cao X. Liu Y. Tang F. R. (2012). CCL28 in the mouse hippocampal CA1 area and the dentate gyrus during and after pilocarpine-induced status epilepticus. Neurochem. Int. 61, 1094–1101. 10.1016/j.neuint.2012.08.001 22917922

[B69] Liva S. M. de Vellis J. (2001). IL-5 induces proliferation and activation of microglia via an unknown receptor. Neurochem. Res. 26, 629–637. 10.1023/A:1010983119125 11523538

[B70] Lok J. Sardi S. P. Guo S. Besancon E. Ha D. M. Rosell A.. (2009). Neuregulin-1 signaling in brain endothelial cells. J. Cereb. Blood Flow Metab. 29, 39–43. 10.1038/jcbfm.2008.94 18728681 PMC3680122

[B71] Luk K. C. Kehm V. Carroll J. Zhang B. O'Brien P. Trojanowski J. Q.. (2012). Pathological α-synuclein transmission initiates Parkinson-like neurodegeneration in nontransgenic mice. Science 338, 949–953. 10.1126/science.1227157 23161999 PMC3552321

[B72] Mahlknecht P. Stemberger S. Sprenger F. Rainer J. Hametner E. Kirchmair R.. (2012). An antibody microarray analysis of serum cytokines in neurodegenerative Parkinsonian syndromes. Proteome Sci. 10:71. 10.1186/1477-5956-10-71 23173604 PMC3539904

[B73] Mandel S. Grunblatt E. Riederer P. Amariglio N. Jacob-Hirsch J. Rechavi G.. (2005). Gene expression profiling of sporadic Parkinson's disease substantia nigra pars compacta reveals impairment of ubiquitin-proteasome subunits, SKP1A, aldehyde dehydrogenase, and chaperone HSC-70. Ann. N.Y. Acad. Sci. 1053, 356–375. 10.1196/annals.1344.031 16179542

[B74] Mangano E. N. Litteljohn D. So R. Nelson E. Peters S. Bethune C.. (2012). Interferon-γ plays a role in paraquat-induced neurodegeneration involving oxidative and proinflammatory pathways. Neurobiol. Aging 33, 1411–1426. 10.1016/j.neurobiolaging.2011.02.016 21482445

[B75] Masada T. Hua Y. Xi G. Yang G. Y. Hoff J. T. Keep R. F.. (2003). Overexpression of interleukin-1 receptor antagonist reduces brain edema induced by intracerebral hemorrhage and thrombin. Acta Neurochir. Suppl. 86, 463–467. 14753487 10.1007/978-3-7091-0651-8_95

[B76] Masuda-Suzukake M. Nonaka T. Hosokawa M. Kubo M. Shimozawa A. Akiyama H.. (2014). Pathological alpha-synuclein propagates through neural networks. Acta Neuropathol. Commun. 2, 88. 10.1186/s40478-014-0088-8 25095794 PMC4147188

[B77] McGeer P. L. Itagaki S. Boyes B. E. McGeer E. G. (1988). Reactive microglia are positive for HLA-DR in the substantia nigra of Parkinson's and Alzheimer's disease brains. Neurology 38, 1285–1291. 10.1212/WNL.38.8.1285 3399080

[B78] McGuire S. O. Ling Z. D. Lipton J. W. Sortwell C. E. Collier T. J. Carvey P. M. (2001). Tumor necrosis factor alpha is toxic to embryonic mesencephalic dopamine neurons. Exp. Neurol. 169, 219–230. 10.1006/exnr.2001.7688 11358437

[B79] Mogi M. Harada M. Kondo T. Riederer P. Nagatsu T. (1996). Interleukin-2 but not basic fibroblast growth factor is elevated in parkinsonian brain. Short communication. J. Neural Transm. 103, 1077–1081. 10.1007/BF01291792 9013395

[B80] Mogi M. Harada M. Riederer P. Narabayashi H. Fujita K. Nagatsu T. (1994). Tumor necrosis factor-alpha (TNF-alpha) increases both in the brain and in the cerebrospinal fluid from parkinsonian patients. Neurosci. Lett. 165, 208–210. 10.1016/0304-3940(94)90746-3 8015728

[B81] Mohapel P. Frielingsdorf H. Häggblad J. Zachrisson O. Brundin P. (2005). Platelet-derived growth factor (PDGF-BB) and brain-derived neurotrophic factor (BDNF) induce striatal neurogenesis in adult rats with 6-hydroxydopamine lesions. Neuroscience 132, 767–776. 10.1016/j.neuroscience.2004.11.056 15837137

[B82] Monahan A. J. Warren M. Carvey P. M. (2008). Neuroinflammation and peripheral immune infiltration in Parkinson's disease: an autoimmune hypothesis. Cell Transplant. 17, 363–372. 10.3727/096368908784423328 18522239

[B83] Montgomery S. L. Bowers W. J. (2012). Tumor necrosis factor-alpha and the roles it plays in homeostatic and degenerative processes within the central nervous system. J. Neuroimmune Pharmacol. 7, 42–59. 10.1007/s11481-011-9287-2 21728035

[B84] Mount M. P. Lira A. Grimes D. Smith P. D. Faucher S. Slack R.. (2007). Involvement of interferon-gamma in microglial-mediated loss of dopaminergic neurons. J. Neurosci. 27, 3328–3337. 10.1523/JNEUROSCI.5321-06.2007 17376993 PMC6672486

[B85] Nagatsu T. Mogi M. Ichinose H. Togari A. (2000a). Changes in cytokines and neurotrophins in Parkinson's disease. J. Neural Transm. Suppl. 60, 277–290. 10.1007/978-3-7091-6301-6_19 11205147

[B86] Nagatsu T. Mogi M. Ichinose H. Togari A. (2000b). Cytokines in Parkinson's disease. J. Neural Transm. Suppl. 58, 143–151. 10.1007/978-3-7091-6284-2_12 11128604

[B87] Ohnuki T. Nakamura A. Okuyama S. Nakamura S. (2010). Gene expression profiling in progressively MPTP-lesioned macaques reveals molecular pathways associated with sporadic Parkinson's disease. Brain Res. 1346, 26–42. 10.1016/j.brainres.2010.05.066 20513370

[B88] Osterberg V. R. Spinelli K. J. Weston L. J. Luk K. C. Woltjer R. L. Unni V. K. (2015). Progressive aggregation of alpha-synuclein and selective degeneration of lewy inclusion-bearing neurons in a mouse model of parkinsonism. Cell Rep. 10, 1252–1260. 10.1016/j.celrep.2015.01.060 25732816 PMC4351119

[B89] Ostrerova-Golts N. Petrucelli L. Hardy J. Lee J. M. Farer M. Wolozin B. (2000). The A53T alpha-synuclein mutation increases iron-dependent aggregation and toxicity. J. Neurosci. 20, 6048–6054. 10934254 10.1523/JNEUROSCI.20-16-06048.2000 PMC6772599

[B90] Ozaki M. Kishigami S. Yano R. (1998). Expression of receptors for neuregulins, ErbB2, ErbB3 and ErbB4, in developing mouse cerebellum. Neurosci. Res. 30, 351–354. 10.1016/S0168-0102(98)00013-3 9678639

[B91] Parkinson's Disease Foundaton (2015). Statistics on Parkinson's. Available online at: http://www.pdf.org/en/parkinson_statistics

[B92] Paul G. Zachrisson O. Varrone A. Almqvist P. Jerling M. Lind G.. (2015). Safety and tolerability of intracerebroventricular PDGF-BB in Parkinson's disease patients. J. Clin. Invest. 125, 1339–1346. 10.1172/JCI79635 25689258 PMC4362250

[B93] Paumier K. L. Luk K. C. Manfredsson F. P. Kanaan N. M. Lipton J. W. Collier T. J.. (2015). Intrastriatal injection of pre-formed mouse α-synuclein fibrils into rats triggers α-synuclein pathology and bilateral nigrostriatal degeneration. Neurobiol. Dis. 82, 185–199. 10.1016/j.nbd.2015.06.003 26093169 PMC4640952

[B94] Pei Z. Pang H. Qian L. Yang S. Wang T. Zhang W.. (2007). MAC1 mediates LPS-induced production of superoxide by microglia: the role of pattern recognition receptors in dopaminergic neurotoxicity. Glia 55, 1362–1373. 10.1002/glia.20545 17654704

[B95] Polymeropoulos M. H. (1997). Mutation in the -synuclein gene identified in families with Parkinson's Disease. Science 276, 2045–2047. 10.1126/science.276.5321.2045 9197268

[B96] Prieto A. L. O'Dell S. Varnum B. Lai C. (2007). Localization and signaling of the receptor protein tyrosine kinase Tyro3 in cortical and hippocampal neurons. Neuroscience 150, 319–334. 10.1016/j.neuroscience.2007.09.047 17980494 PMC2231337

[B97] Qin L. Liu Y. Hong J.-S. Crews F. T. (2013). NADPH oxidase and aging drive microglial activation, oxidative stress, and dopaminergic neurodegeneration following systemic LPS administration. Glia 61, 855–868. 10.1002/glia.22479 23536230 PMC3631289

[B98] Ringheim G. E. (1995). Mitogenic effects of interleukin-5 on microglia. Neurosci. Lett. 201, 131–134. 10.1016/0304-3940(95)12153-6 8848235

[B99] Rocha N. P. Teixeira A. L. Scalzo P. L. Barbosa I. G. de Sousa M. S. Morato I. B.. (2014). Plasma levels of soluble tumor necrosis factor receptors are associated with cognitive performance in Parkinson's disease. Mov. Disord. 29, 527–531. 10.1002/mds.25752 24301904

[B100] Sanderson K. L. Raghupathi R. Saatman K. E. Martin D. Miller G. McIntosh T. K. (1999). Interleukin-1 receptor antagonist attenuates regional neuronal cell death and cognitive dysfunction after experimental brain injury. J. Cereb. Blood Flow Metab. 19, 1118–1125. 10.1097/00004647-199910000-00008 10532636

[B101] Sawada M. Suzumura A. Itoh Y. Marunouchi T. (1993). Production of interleukin-5 by mouse astrocytes and microglia in culture. Neurosci. Lett. 155, 175–178. 10.1016/0304-3940(93)90701-L 8377948

[B102] Sharif A. Duhem-Tonnelle V. Allet C. Baroncini M. Loyens A. Kerr-Conte J.. (2009). Differential erbB signaling in astrocytes from the cerebral cortex and the hypothalamus of the human brain. Glia 57, 362–379. 10.1002/glia.20762 18803307

[B103] Sharma N. Nehru B. (2015). Characterization of the lipopolysaccharide induced model of Parkinson's disease: role of oxidative stress and neuroinflammation. Neurochem. Int. 87, 92–105. 10.1016/j.neuint.2015.06.004 26055970

[B104] Sian-Hulsmann J. Monoranu C. Strobel S. Riederer P. (2015). Lewy bodies: a spectator or salient killer? CNS Neurol. Disord. Drug Targets 14, 947–955. 10.2174/1871527314666150317225659 25801839

[B105] Smits A. Odin P. Duan W. M. Brundin P. Widner H. Heldin C. H.. (1993). Expression of platelet-derived growth factor in and around intrastriatal embryonic mesencephalic grafts. Cell Transplant. 2, 151–162. 7908247 10.1177/096368979300200208

[B106] Stefanova N. Fellner L. Reindl M. Masliah E. Poewe W. Wenning G. K. (2011). Toll-like receptor 4 promotes α-synuclein clearance and survival of nigral dopaminergic neurons. Am. J. Pathol. 179, 954–963. 10.1016/j.ajpath.2011.04.013 21801874 PMC3157205

[B107] Steiner H. Blum M. Kitai S. T. Fedi P. (1999). Differential expression of ErbB3 and ErbB4 neuregulin receptors in dopamine neurons and forebrain areas of the adult rat. Exp. Neurol. 159, 494–503. 10.1006/exnr.1999.7163 10506520

[B108] Stone K. P. Kastin A. J. Pan W. (2011). NF?B is an unexpected major mediator of interleukin-15 signaling in cerebral endothelia. Cell. Physiol. Biochem. 28, 115–124. 10.1159/000331720 21865854 PMC3709181

[B109] Sulpice E. Bryckaert M. Lacour J. Contreres J.-O. Tobelem G. (2002). Platelet factor 4 inhibits FGF2-induced endothelial cell proliferation via the extracellular signal-regulated kinase pathway but not by the phosphatidylinositol 3-kinase pathway. Blood 100, 3087–3094. 10.1182/blood.V100.9.3087 12384403

[B110] Tanaka S. Ishii A. Ohtaki H. Shioda S. Yoshida T. Numazawa S. (2013). Activation of microglia induces symptoms of Parkinson's disease in wild-type, but not in IL-1 knockout mice. J. Neuroinflammation 10:143. 10.1186/1742-2094-10-143 24289537 PMC4220804

[B111] Tansey M. G. Frank-Cannon T. C. McCoy M. K. Lee J. K. Martinez T. N. McAlpine F. E.. (2008). Neuroinflammation in Parkinson's disease: is there sufficient evidence for mechanism-based interventional therapy? Front. Biosci. 13:709–717. 10.2741/2713 17981581

[B112] Tansey M. G. McCoy M. K. Frank-Cannon T. C. (2007). Neuroinflammatory mechanisms in Parkinson's disease: potential environmental triggers, pathways, and targets for early therapeutic intervention. Exp. Neurol. 208, 1–25. 10.1016/j.expneurol.2007.07.004 17720159 PMC3707134

[B113] Taylor J. M. Main B. S. Crack P. J. (2013). Neuroinflammation and oxidative stress: co-conspirators in the pathology of Parkinson's disease. Neurochem. Int. 62, 803–819. 10.1016/j.neuint.2012.12.016 23291248

[B114] Tran T. A. Nguyen A. D. Chang J. Goldberg M. S. Lee J.-K. Tansey M. G. (2011). Lipopolysaccharide and tumor necrosis factor regulate Parkin expression via nuclear factor-kappa B. PLoS ONE 6:e23660. 10.1371/journal.pone.0023660 21858193 PMC3157435

[B115] Vecil G. G. Larsen P. H. Corley S. M. Herx L. M. Besson A. Goodyer C. G.. (2000). Interleukin-1 is a key regulator of matrix metalloproteinase-9 expression in human neurons in culture and following mouse brain trauma *in vivo*. J. Neurosci. Res. 61, 212–224. 10.1002/1097-4547(20000715)61:2%3C212::AID-JNR12%3E3.0.CO;2-9 10878594

[B116] Vekrellis K. Stefanis L. (2012). Targeting intracellular and extracellular alpha-synuclein as a therapeutic strategy in Parkinson's disease and other synucleinopathies. Expert Opin. Ther. Targets 16, 421–432. 10.1517/14728222.2012.674111 22480256

[B117] Vivekanantham S. Shah S. Dewji R. Dewji A. Khatri C. Ologunde R. (2015). Neuroinflammation in Parkinson's disease: role in neurodegeneration and tissue repair. Int. J. Neurosci. 125, 717–725. 10.3109/00207454.2014.982795 25364880

[B118] Walker D. G. Dalsing-Hernandez J. E. Campbell N. A. Lue L.-F. (2009). Decreased expression of CD200 and CD200 receptor in Alzheimer's disease: a potential mechanism leading to chronic inflammation. Exp. Neurol. 215, 5–19. 10.1016/j.expneurol.2008.09.003 18938162 PMC2765462

[B119] Walker D. G. Whetzel A. M. Lue L.-F. (2015a). Expression of suppressor of cytokine signaling genes in human elderly and Alzheimer's disease brains and human microglia. Neuroscience 302, 121–137. 10.1016/j.neuroscience.2014.09.052 25286386 PMC4385752

[B120] Walker D. G. Whetzel A. M. Serrano G. Sue L. I. Beach T. G. Lue L.-F. (2015b). Association of CD33 polymorphism rs3865444 with Alzheimer's disease pathology and CD33 expression in human cerebral cortex. Neurobiol. Aging 36, 571–582. 10.1016/j.neurobiolaging.2014.09.023 25448602 PMC4315751

[B121] Watson M. B. Richter F. Lee S. K. Gabby L. Wu J. Masliah E.. (2012). Regionally-specific microglial activation in young mice over-expressing human wildtype alpha-synuclein. Exp. Neurol. 237, 318–334. 10.1016/j.expneurol.2012.06.025 22750327 PMC3443323

[B122] Wilms H. Rosenstiel P. Sievers J. Deuschl G. Zecca L. Lucius R. (2003). Activation of microglia by human neuromelanin is NF-kappaB dependent and involves p38 mitogen-activated protein kinase: implications for Parkinson's disease. FASEB J. 17, 500–502. 10.1096/fj.02-0314fje 12631585

[B123] Yang G. Y. Mao Y. Zhou L. F. Gong C. Ge H. L. Betz A. L. (1999). Expression of intercellular adhesion molecule 1 (ICAM-1) is reduced in permanent focal cerebral ischemic mouse brain using an adenoviral vector to induce overexpression of interleukin-1 receptor antagonist. Brain Res. Mol. Brain Res. 65, 143–150. 10.1016/S0169-328X(98)00335-0 10064885

[B124] Yasuhara O. Matsuo A. Terai K. Walker D. G. Berger A. E. Akiguchi I.. (1997). Expression of interleukin-1 receptor antagonist protein in post-mortem human brain tissues of Alzheimer's disease and control cases. Acta Neuropathol. 93, 414–420. 10.1007/s004010050633 9113207

[B125] Zhang W. Wang T. Pei Z. Miller D. S. Wu X. Block M. L.. (2005). Aggregated alpha-synuclein activates microglia: a process leading to disease progression in Parkinson's disease. FASEB J. 19, 533–542. 10.1096/fj.04-2751com 15791003

[B126] Zhang W. Zecca L. Wilson B. Ren H.-W. Wang Y.-J. Wang X.-M.. (2013). Human neuromelanin: an endogenous microglial activator for dopaminergic neuron death. Front. Biosci. (Elite Ed). 5:1–11. 10.2741/E591 23276965 PMC3626451

[B127] Zhao C. Ling Z. Newman M. B. Bhatia A. Carvey P. M. (2007). TNF-alpha knockout and minocycline treatment attenuates blood-brain barrier leakage in MPTP-treated mice. Neurobiol. Dis. 26, 36–46. 10.1016/j.nbd.2006.11.012 17234424 PMC1892817

[B128] Zhao Q. M. Li L. M. Zhang C. Guo R. (2012). Expression and localization of receptor tyrosine kinase Tyro3 in rat brain. Beijing Da Xue Xue Bao. 44, 905–910. 23247456

[B129] Zheng Y. Wang Q. Xiao B. Lu Q. Wang Y. Wang X. (2012). Involvement of receptor tyrosine kinase Tyro3 in amyloidogenic APP processing and β-amyloid deposition in Alzheimer's disease models. PLoS ONE 7:e39035. 10.1371/journal.pone.0039035 22701746 PMC3372537

