# Supplementary material for: Altered Expression Patterns of Inflammation-Associated and Trophic Molecules in Substantia Nigra and Striatum Brain Samples from Parkinson's Disease, Incidental Lewy Body Disease and Normal Control Cases
Source: Front Neurosci. 2016 Jan 14;9:507. doi: 10.3389/fnins.2015.00507 (PMC4712383; doi:10.3389/fnins.2015.00507)
Supplement: Supplementary file 1 [file Table1.DOCX]

**Supplemental Table 1. (Definition/Abbreviations of Array Features)**

| **ARRAY-CHE** | |
| --- | --- |
| 6Ckine | Chemokine (C-C motif) ligand 21 (CCL21) |
| Axl | Tyrosine-protein kinase receptor UFO |
| BTC | Betacellulin |
| CCL28 | Mucosae-associated epithelial chemokine (MEC) |
| CTACK | Cutaneous T-cell attracting chemokine |
| CXCL16 | Chemokine (C-X-C motif) ligand 16 |
| ENA-78 | C-X-C motif chemokine 5 |
| Eotaxin-3 | Eosinophil chemotactic protein-3 (CCL26) |
| GCP-2 | Granulocyte chemotactic protein 2 (CXCL6) |
| GRO | Chemokine (C-X-C motif) ligand 1 |
| HCC-1 | Chemokine (C-C motif) ligand 14 (CCL14) |
| HCC-4 | Chemokine (C-C motif) ligand 16 (CCL16) |
| IL-9 | Interleukin-9 |
| IL-17F | Interleukin-17 receptor A |
| IL-18 BPa | Interleukin-18-binding protein alpha |
| IL-28A | Interleukin-28 receptor |
| IL-29 | Interleukin-29 |
| IL-31 | Interleukin-31 |
| IP-10 | Interferon gamma-induced protein 10 (CXC10) |
| I-TAC | Interferon-inducible T-cell alpha chemoattractant |
| LIF | Leukemia inhibitory factor |
| LIGHT | Homologous to lymphotoxin, |
| Ltactin | Lymphotactin (XCL1) |
| MCP-2 | Monocyte chemoattractant protein 2 (CCL8) |
| MCP-3 | Monocyte chemoattractant protein 3 (CCL7) |
| MCP-4 | Monocyte chemoattractant protein 3 (CCL13) |
| MDC | Macrophage-derived chemokine (CCL22) |
| MIF | Macrophage migration inhibitory factor |
| MIP-3a | Macrophage Inflammatory Protein 3-alpha (CCL20) |
| MIP-3b | Macrophage Inflammatory Protein 3-beta (CCL19) |
| MPIF-1 | Myeloid progenitor inhibitory factor 1 (CCL23) |
| MSPa | Mycobacterial porin |
| NAP-2 | Nucleosome Assembly protein 2 |
| OPN | Osteopontin |
| PARC | Pulmonary and activation-regulated chemokine (CCL18) |
| PF4 | Platelet factor 4 |
| SDF-1a | Stromal cell-derived factor 1-alpha (CXCL12) |
| TARC | Thymus and activation regulated chemokine (CCL17) |
| TECK | Thymus-expressed chemokine (CCL25) |
| TSLP | Thymic stromal lymphopoietin |

| **ARRAY-CYT** | |
| --- | --- |
| BLC | B-lymphocyte chemoattractant(CXCL13) |
| Eotaxin | Eosinophil chemotactic protein (CCL11) |
| Eotaxin-2 | Eosinophil chemotactic protein-2 (CCL24) |
| G-CSF | Granulocyte colony-stimulating factor |
| GM-CSF | Granulocyte macrophage colony-stimulating factor |
| I-309 | Chemokine (C-C motif) ligand 1 (CCL1) |
| ICAM-1 | Intercellular adhesion molecule 1 |
| IFNγ | Interferon gamma |
| IL-1α | Interleukin-1 alpha |
| IL-1β | Interleukin-1 beta |
| IL-1ra | Interleukin-1 receptor antagonist |
| IL-2 | Interleukin-2 |
| IL-4 | Interleukin-4 |
| IL-5 | Interleukin-5 |
| IL-6 | Interleukin-6 |
| IL-6sR | Interleukin-6 receptor, soluble |
| IL-7 | Interleukin-7 |
| IL-8 | Interleukin-8 |
| IL-10 | Interleukin-10 |
| IL-11 | Interleukin-11 |
| IL-12p40 | Interleukin-12p40 |
| IL-12p70 | Interleukin-12p70 |
| IL-13 | Interleukin-13 |
| IL-15 | Interleukin-15 |
| IL-16 | Interleukin-16 |
| IL-17 | Interleukin-17 |
| MCP-1 | Monocyte chemotactic protein 1 (CCL2) |
| MCSF | Macrophage colony-stimulating factor |
| MIG | Monokine induced by gamma interferon |
| MIP-1a | Macrophage inflammatory protein-1 alpha (CCL3) |
| MIP-1b | Macrophage inflammatory protein-1 beta (CCL4) |
| MIP1d | Macrophage inflammatory protein-1 delta (CCL15) |
| PDGF-BB | Platelet-derived growth factor subunit BB |
| RANTES | Regulated on activation, normal T cell expressed and secreted (CCL5) |
| TIMP-1 | Tissue inhibitor of metalloproteinases-1 |
| TIMP-2 | Tissue inhibitor of metalloproteinases-1 |
| TNFα | Tumor necrosis factor-alpha |
| TNFβ | Tumor necrosis factor-beta |
| TNF RI | Tumor necrosis factor receptor-1 |
| TNF RII | Tumor necrosis factor-beta receptor-2 |

| **ARRAY-REC** | |
| --- | --- |
| 4-1BB | CD137 |
| ALCAM | Activated leukocyte cell adhesion molecule |
| B7-1 | CD80 |
| BCMA | B-Cell Maturation Antigen |
| CD14 | Cluster of differentiation 14 |
| CD30 | Cluster of differentiation 30 (TNFRSF8) |
| CD40 L | Cluster of differentiation 40 Ligand (CD154) |
| CEACAM-1 | Carcinoembryonic antigen-related cell adhesion molecule 1 (biliary glycoprotein) |
| DR6 | Death receptor 6 |
| Dtk | TYRO3 protein tyrosine kinase receptor |
| Endoglin | CD105 |
| ErbB3 | Receptor tyrosine-protein kinase erb B-3 |
| E-Selectin | CD-62 antigen-like family member E |
| Fas | Fas cell surface death receptor (APO-1) |
| Flt-3L | FMS-like tyrosine kinase 3 ligand |
| GITR | Glucocorticoid-induced TNFR-related protein |
| HVEM | Herpesvirus entry mediator |
| ICAM-3 | Intercellular adhesion molecule 3 |
| IL-1 R4 | Interleukin-1 receptor type 4 |
| IL-1 R1 | Interleukin-1 receptor type 1 |
| IL-2 Rg | Interleukin-2 receptor subunit gamma |
| IL-10 Rb | Interleukin-10 receptor subunit beta |
| IL-17R | Interleukin-17 receptor |
| IL-21R | Interleukin-21 receptor |
| LIMPII | Lysosomal integral membrane protein II |
| Lipocalin-2 | Lipocalin-2 (NGAL) |
| L-selectin | L-selectin (CD62L) |
| LYVE-1 | Lymphatic vessel endothelial hyaluronan receptor 1 |
| MICA | MHC class I polypeptide-related sequence A |
| MICB | MHC class I polypeptide-related sequence B |
| NRG1-b1 | Neuregulin-1 beta-1 |
| PDGF Rb | Platelet-derived growth factor receptor subunit beta |
| PECAM-1 | Platelet/endothelial cell adhesion molecule 1 (CD31) |
| RAGE | Advanced glycosylation end product-specific receptor |
| TIM-1 | T-cell immunoglobulin and mucin domain 1 (HAVcr-1) |
| TRAIL R3 | Receptor for the cytotoxic ligand TRAIL |
| Trappin-2 | \| Peptidase inhibitor 3 \| \| --- \| |
| uPAR | Urokinase plasminogen activator receptor |
| VCAM-1 | Vascular cell adhesion molecule 1 |
| XEDAR | X-linked ectodermal dysplasia receptor |

| ARRAY-GF | |
| --- | --- |
| AR | Androgen receptor |
| BDNF | Brain-derived neurotrophic factor |
| bFGF | Basic fibroblast growth factor |
| BMP-4 | Bone morphogenetic protein 4 |
| BMP-5 | Bone morphogenetic protein 5 |
| BMP-7 | Bone morphogenetic protein 7 |
| b-NGF | Beta nerve growth factor |
| EGF | Epidermal growth factor |
| EGF-R | Epidermal growth factor receptor |
| EG-VEGF | Endocrine gland-derived vascular endothelial growth factor |
| FGF-4 | Fibroblast growth factor 4 |
| FGF-7 | Fibroblast growth factor 7 (KGF) |
| GDF-15 | Growth differentiation factor 15 |
| GDNF | Glial cell derived neurotrophic factor |
| GH | Growth hormone |
| HB-EGF | Heparin-binding EGF-like growth factor |
| HGF | Hepatocyte growth factor |
| IGFBP-1 | Insulin-like growth factor-binding protein 1 |
| IGFBP-2 | Insulin-like growth factor-binding protein 2 |
| IGFBP-3 | Insulin-like growth factor-binding protein 3 |
| IGFBP-4 | Insulin-like growth factor-binding protein 4 |
| IGFBP-6 | Insulin-like growth factor-binding protein 6 |
| IGF-1 | Insulin-like growth factor 1 |
| Insulin | Insulin |
| MCF R | Mitochondrial substrate carrier family protein R |
| NGF R | Nerve growth factor receptor |
| NT-3 | Neurotrophin-3 |
| NT-4 | Neurotrophin-4 |
| OPG | Osteoprotegerin, osteoclastogenesis inhibitory factor |
| PDGF-AA | Platelet-derived growth factor-AA |
| PIGF | Phosphatidylinositol-glycan biosynthesis class F protein |
| SCF | Stem cell factor |
| SCF R | Stem cell factor receptor, cKIT |
| TGFa | Transforming growth factor alpha |
| TGFb1 | Transforming growth factor beta 1 |
| TGFb3 | Transforming growth factor beta-3 |
| VEGF | Vascular endothelial growth factor |
| VEGF R2 | Vascular endothelial growth factor receptor 2 |
| VEGF R3 | Vascular endothelial growth factor receptor 3 |
| VEGF-D | Vascular endothelial growth factor D |
